# Transcriptional Profiling of the Oral Pathogen *Streptococcus mutans* in Response to Competence Signaling Peptide XIP

**DOI:** 10.1128/mSystems.00102-16

**Published:** 2017-01-03

**Authors:** Iwona B. Wenderska, Andrew Latos, Benjamin Pruitt, Sara Palmer, Grace Spatafora, Dilani B. Senadheera, Dennis G. Cvitkovitch

**Affiliations:** a Dental Research Institute, Faculty of Dentistry, University of Toronto, Toronto, Ontario, Canada; b Department of Biology, Middlebury College, Middlebury, Vermont, USA; c College of Dentistry, Biosciences, Ohio State University, Columbus, Ohio, USA; Leiden University

**Keywords:** ComRS, *Streptococcus mutans*, XIP, cell signaling, genetic competence, signaling peptides, transcriptome

## Abstract

In the cariogenic *Streptococcus mutans*, competence development is regulated by the ComRS signaling system comprised of the ComR regulator and the ComS prepeptide to the competence signaling peptide XIP (ComX-inducing peptide). Aside from competence development, XIP signaling has been demonstrated to regulate cell lysis, and recently, the expression of bacteriocins, small antimicrobial peptides used by bacteria to inhibit closely related species. Our study further explores the effect of XIP signaling on the *S. mutans* transcriptome. RNA sequencing revealed that XIP induction resulted in a global change in gene expression that was consistent with a stress response. An increase in several membrane-bound regulators, including HdrRM and BrsRM, involved in bacteriocin production, and the VicRKX system, involved in acid tolerance and biofilm formation, was observed. Furthermore, global changes in gene expression corresponded to changes observed during the stringent response to amino acid starvation. Effects were also observed on genes involved in sugar transport and carbon catabolite repression and included the *levQRST* and *levDEFG* operons. Finally, our work identified a novel heat shock-responsive intergenic region, encoding a small RNA, with a potential role in competence shutoff.

**IMPORTANCE** Genetic competence provides bacteria with an opportunity to increase genetic diversity or acquire novel traits conferring a survival advantage. In the cariogenic pathogen *Streptococcus mutans*, DNA transformation is regulated by the competence stimulating peptide XIP (ComX-inducing peptide). The present study utilizes high-throughput RNA sequencing (RNAseq) to provide a greater understanding of how global gene expression patterns change in response to XIP. Overall, our work demonstrates that in *S. mutans*, XIP signaling induces a response that resembles the stringent response to amino acid starvation. We further identify a novel heat shock-responsive intergenic region with a potential role in competence shutoff. Together, our results provide further evidence that multiple stress response mechanisms are linked through the genetic competence signaling pathway in *S. mutans*.

## Abstract (precis)

Genetic competence provides bacteria with an opportunity to increase genetic diversity or acquire novel traits conferring a survival advantage. In the cariogenic pathogen *Streptococcus mutans*, DNA transformation is regulated by the competence stimulating peptide XIP (ComX-inducing peptide). The present study utilizes high-throughput RNA sequencing (RNAseq) to provide a greater understanding of how global gene expression patterns change in response to XIP. Overall, our work demonstrates that in *S. mutans*, XIP signaling induces a response that resembles the stringent response to amino acid starvation. We further identify a novel heat shock-responsive intergenic region with a potential role in competence shutoff. Together, our results provide further evidence that multiple stress response mechanisms are linked through the genetic competence signaling pathway in *S. mutans*.

## INTRODUCTION

The ability to take up and incorporate extracellular DNA (eDNA) allows bacteria to modify their genomes, leading to an increase in genetic variability and population survival under changing environmental conditions (1). Aside from its role in genetic diversity, the process of transformation by genetically competent cells has been suggested to play a role in utilization of eDNA as a nutrient or as a template for DNA repair of chromosomal damage (2, 3). In fact, genetic competence has been suggested to function as part of a general stress response mechanism in many Gram-positive bacteria (4).

In streptococci, the process of transformation is often regulated by peptide signaling mechanisms that drive the expression of an alternate sigma factor *comX*, which, in turn, tightly regulates the expression of the DNA uptake and recombination machinery (5). In *Streptococcus mutans*, a caries-causing oral pathogen, genetic competence is regulated by the ComRS signaling system, comprised of the ComS prepeptide to the ComX-inducing peptide XIP and its cognate response regulator ComR (6). The ComS prepeptide is secreted out of the cell and processed by an unknown mechanism. Upon import by the OppD/Ami permease, the mature XIP interacts with and activates ComR to directly regulate the expression of *comX* (6, 7). The ComR-XIP complex also binds to the promoter of the *comS* peptide, creating a positive-feedback loop (6, 7). Our recent observation that a *comX* deletion strain contains significantly less XIP in the supernatant suggests a second positive-feedback loop that allows the ComX sigma factor to regulate XIP production and/or secretion (8). Aside from its essential role in competence regulation, ComRS and ComX also regulate lysis in a subpopulation of cells (8). A ComX-regulated lysin, LytF, has recently been identified as a contributor to competence-associated cell lysis (9). This mechanism ensures the availability of DNA for genetic exchange, nutrition, or chromosome repair during the stress-activated state of competence.

Recent work demonstrated that XIP signaling also regulates the expression of the ComDE two-component system (TCS) that regulates the bacteriocin genes (10), which encode small antimicrobial peptides that inhibit the growth of closely related microbial species. The ComDE TCS is encoded by genes in an operon with the gene encoding the ComC prepeptide, which is processed to the competence-stimulating peptide CSP. The ComDE system directly regulates several bacteriocin genes in response to CSP and plays an indirect role in the activation of competence via ComRS (11, 12). The tight association of competence development and bacteriocin production may provide *S. mutans* with a mechanism to lyse its competitors and simultaneously utilize their genetic contents to increase genome plasticity (10).

Aside from its role in competence and cell lysis, little is known regarding the role of XIP signaling in *S. mutans* physiology. Herein we employed strand-specific RNA sequencing (high-throughput RNA sequencing [RNAseq]) to determine the effect of XIP treatment on the whole transcriptome of *S. mutans* UA159. As expected, we observed a large increase in competence-associated genes, the *comX*-regulated lysin LytF, and a large number of the bacteriocin genes. In addition to the known modulation of ComDE, we identified a novel role for XIP signaling to control HdrRM, BrsRM, and VicRKX signaling systems. These regulators function in parallel with the ComDE system to control competence and/or bacteriocin production (13–19). We also observed an increase in the expression of genes involved in nutrient stress, including the *relQ* (p)ppGpp synthase gene, which is involved in the stringent response, and the LevQRST four-component system important to sugar metabolism and carbon catabolite repression. We further report the discovery of 11 intergenic regions that expressed putative short RNAs (sRNAs) and provided the first evidence for a heat shock-regulated intergenic region that negatively regulates genetic competence.

## RESULTS

### Transcriptome changes in *S. mutans* UA159 in response to XIP.

**FIG 1:**
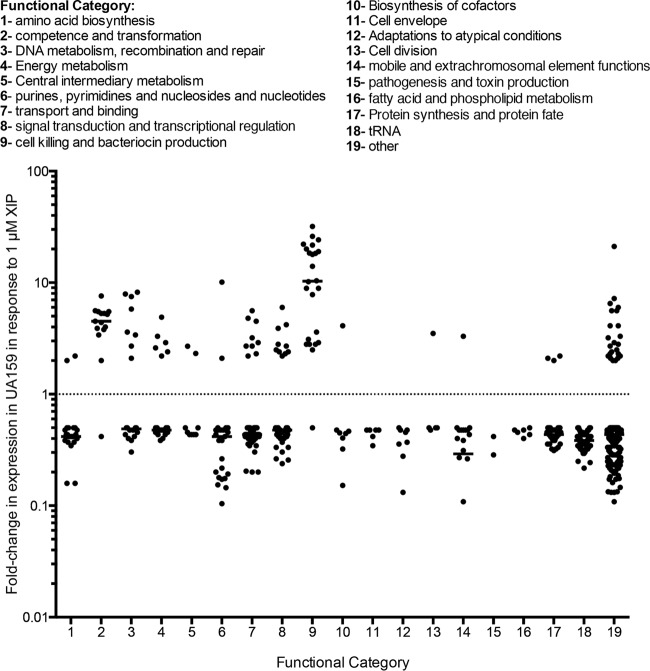
Functional characterization of the changes (>2-fold) in the *S. mutans* UA159 transcriptome in response to 1 µM XIP. Genes up- or downregulated by >2-fold were grouped on the basis of their annotated function, and their fold change values were graphed.

**FIG 2:**
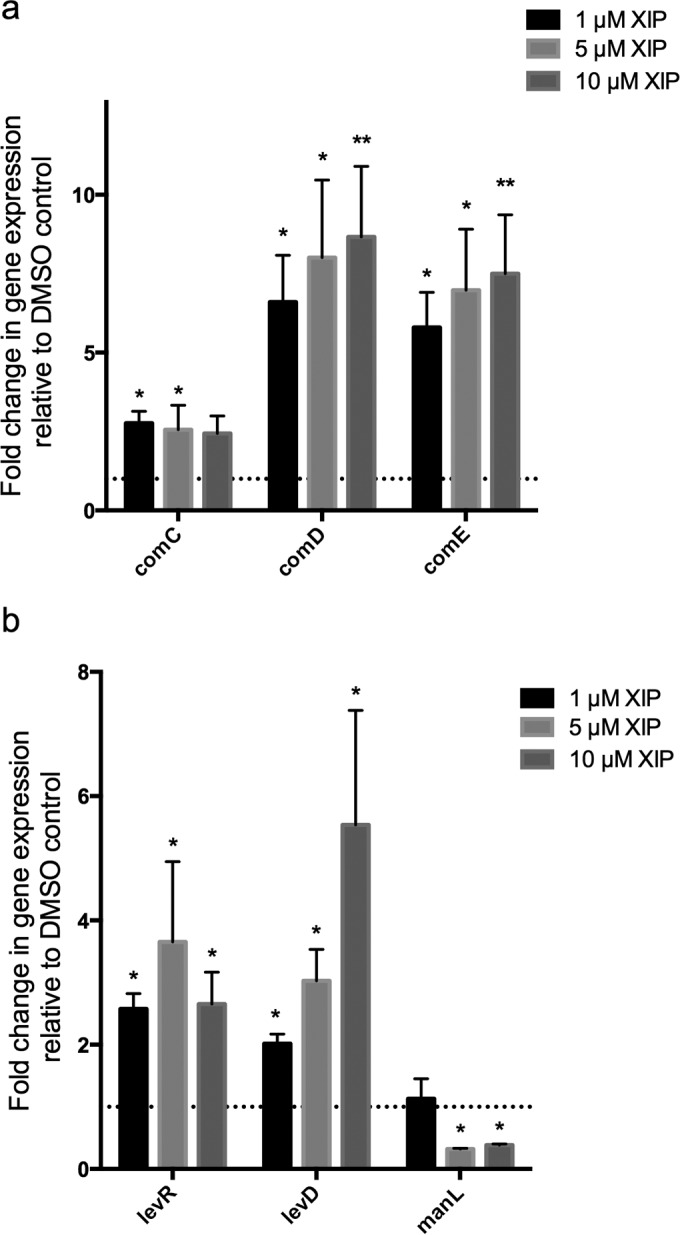
Effects of increasing amounts of XIP on genes involved in bacteriocin regulation (a) and sugar uptake (b). RT-PCR analysis of *comC*, *comD*, and* comE* (a) or *levR*,* levRD*, and *manL* (b) gene expression was performed in strain UA159 grown in CDM to an OD_600 _of 0.4 in the presence of increasing amounts of XIP. Values that are significantly different from the value for the DMSO control are indicated by asterisks as follows: *, *P* < 0.05; **, *P* < 0.005.

To determine the global effects of XIP signaling, we examined the effects of the peptide on the transcriptome of *S. mutans* UA159 using high-throughput RNA sequencing (RNAseq). The cells were grown in chemically defined medium (CDM) to an optical density at 600 nm (OD_600_) of 0.4 to 0.5 in the presence of either 1 µM XIP or the 1% dimethyl sulfoxide (DMSO) vehicle control. Overall, exposure to XIP resulted in the upregulation of 105 genes by greater than twofold in the wild-type (WT) strain (Fig. 1; see Table S2 in the supplemental material). Of these genes, approximately 14 genes were involved in genetic competence and transformation, 8 genes were involved in DNA metabolism, recombination, and repair, and 10 genes were involved in signal transduction and transcriptional regulation. As expected, this list includes the central competence regulator *comX* (4.4-fold), directly regulated by the ComR-XIP complex, as well as many of the ComX-regulated late competence genes involved in DNA uptake and recombination. The strongest upregulation was observed in the 23 genes involved in cell killing and bacteriocin production, including the ComCDE signaling system (2.9-, 2.8- and 3.6-fold, respectively) that directly regulates bacteriocin expression, and the SMU.166-SMU.168 (SMU.166-168) operon (2.8-, 3.1-, and 4.4-fold), proposed to encode a putative three-component toxin-antitoxin system, comprised of the toxin-antitoxin complex and their transcriptional regulator, respectively (20). Upregulation was also observed in 6 genes involved in energy metabolism, 9 genes in transport and binding, and 23 genes with unknown functions. The XIP peptide caused repression of 580 genes in *S. mutans* UA159, 22 of which were involved in amino acid biosynthesis, 23 encoding purines, pyrimidines, nucleosides, and nucleotides, 85 genes involved in protein synthesis, 53 encoding tRNA, and 199 genes with unknown functions (Table S2). Quantitative real-time PCR (qRT-PCR) was used to confirm the role of XIP in the regulation of the bacteriocin regulatory system *comDE*, as previously reported by Reck et al. (10), but also in the regulation of the *comC* gene (Fig. 2a).

Notably, XIP exposure increased the expression of the *levQRST* four-component signaling system (2.3-fold, 2.4-fold, 2.7-fold, and 2.7-fold, respectively), comprised of the LevRS two-component system and two sugar-binding proteins LevQ and LevT, responsible for sensing fructose levels in the extracellular milieu (19, 21). The *levDEFG* operon encoding components of enzyme II of the fructose/mannose phosphotransferase (PTS) system was also upregulated 2.2-, 2.5-, 2.3-, and 2.4-fold, respectively. The LevDEFG is primarily involved in fructose uptake and is upregulated by the LevQRST signaling system in response to low fructose levels (19, 21). Three transcripts corresponding to enzyme II components of the mannose PTS system required for transporting glucose, mannose, sucrose, or cellobiose were downregulated (−4.2-fold, −3.8-fold, and −3.9-fold for *manN*, *manL*, and *manM*, respectively). These components, including *manL*, are important for carbon catabolite repression of the* levDEFG* operon (22). Therefore, the low expression of *manL* could partly explain the increase in *levDEFG* expression. The effects of XIP on representative genes of the *lev* operons and the *manL* gene were confirmed by qRT-PCR (Fig. 2b).

### Changes in gene expression in the ΔSMcomS strain in response to 1 µM XIP.

**FIG 3:**
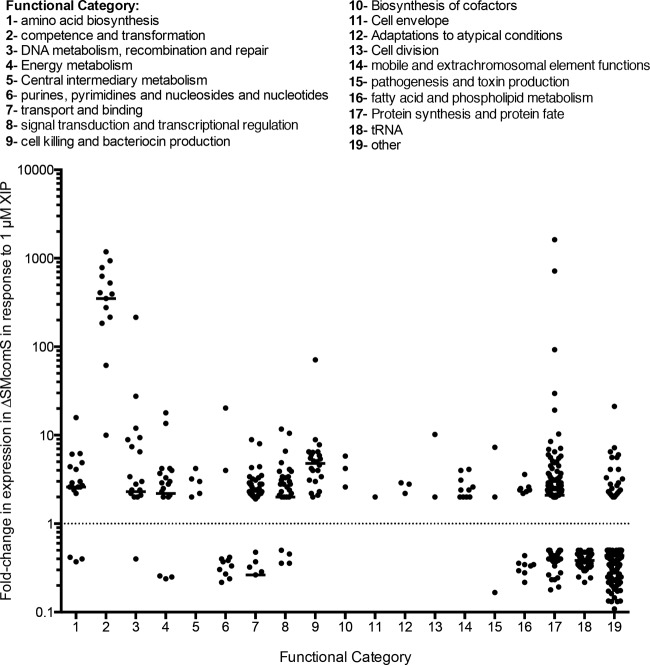
Functional characterization of the changes (>2-fold) in the *S*. *mutans* ΔSMcomS transcriptome in response to 1 µM XIP. Genes up- or downregulated by >2-fold were grouped on the basis of their annotated function, and their fold change values were graphed.

Although genetic transformation in *S. mutans* UA159 does not occur until later stages of growth in CDM, we wanted to ensure that any endogenous XIP production in mid-logarithmic WT cells did not mask any effects on the *S. mutans* transcriptome. As a result, we examined the transcriptome of the *comS* deletion strain that does not produce any endogenous XIP. Treatment of the ΔSMcomS strain with 1 µM XIP resulted in increased expression in almost 300 genes (Fig. 3; Table S3). Of these genes, the highest upregulation was observed in genes involved in competence and transformation. The central competence regulator *comX* was upregulated by 61.4-fold, whereas the genes encoding components of the DNA uptake machinery were upregulated as much as 1,000-fold.

Aside from competence-related genes, several genes involved in virulence were upregulated in the ΔSMcomS strain in response to 1 µM XIP. These genes included genes encoding ABC transporters with predicted involvement in multidrug tolerance and genes involved in adaptation to atypical conditions and detoxification, including the *relQ* synthase gene (2.8-fold) of (p)ppGpp; a mediator of the stringent response that reduces cell growth in response to nutrient starvation (23). Consistent with the stringent response, we also observed a downregulation in genes involved in the synthesis of purines, pyrimidines, nucleosides, and nucleotides. A purine operon repressor, SMU.356, was upregulated 2.1-fold and likely contributed to the low expression of its target genes. Genes involved in pathogenesis, toxin production, and resistance were also upregulated and included several genes involved in mutanobactin production (SMU.1335c-1340), a CSP-regulated secondary metabolite implicated in interkingdom competition with *Candida albicans* (24). In addition, at least a twofold increase was observed in 10 genes encoding transposases and other mobile elements.

**FIG 4:**
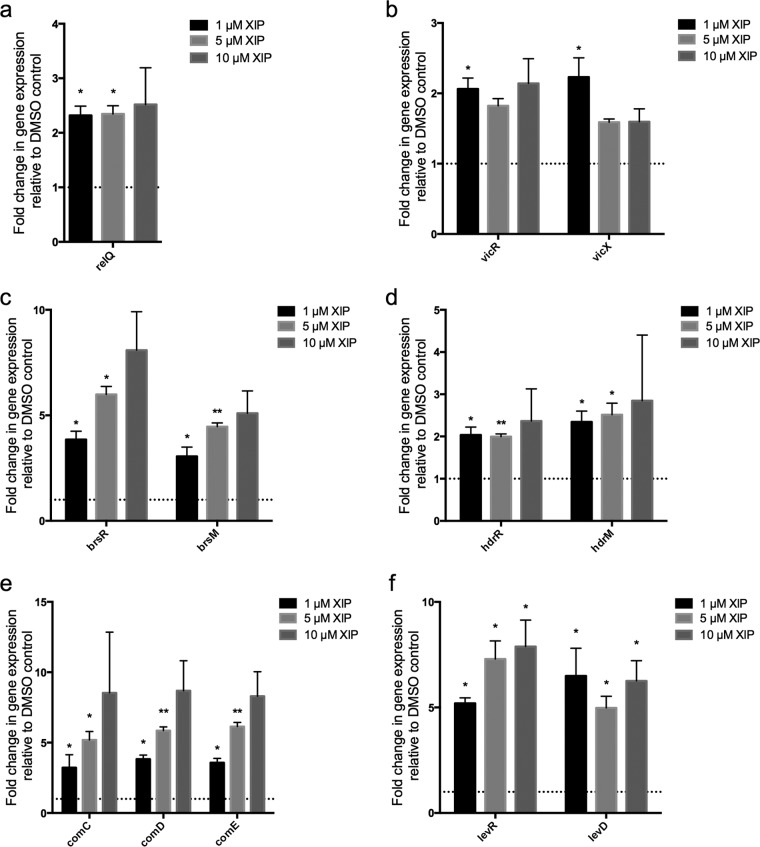
Expression of selected genes in the ΔSMcomS strain in response to increasing concentrations of XIP**.** RT-PCR analysis of gene expression was performed in ΔSMcomS grown in CDM to an OD_600 _of 0.4 in the presence of increasing amounts of XIP. *, *P* < 0.05; **, *P* < 0.005.

A number of signal transduction and transcriptional regulators were also increased in response to XIP treatment, and included the *vicRKX* signaling system (2.0-, 2.0- and 2.4-fold, respectively), strongly associated with virulence of *S. mutans* (16–18), and the *hdrRM* (1.5- and 2.1-fold, respectively) and *brsRM* (5.0- and 4.2-fold, respectively) signaling systems, both of which have previously been demonstrated to function upstream of ComX to regulate competence development and bacteriocin production (13, 14). The *levQRST* system was upregulated 3.5-, 3.9-, 4.1-, and 4.3-fold, respectively, along with its associated fructose/mannose phosphotransferase system, *levDEFG*, which was increased by 3.4-, 3.0-, 2.8-, and 3.3-fold, respectively. The effects of XIP on these membrane-bound regulators of bacteriocin production, sugar metabolism, and virulence expression were confirmed by examining one or more genes representative of each operon by qRT-PCR (Fig. 4).

### Putative small RNAs expressed in the presence of XIP.

**FIG 5:**
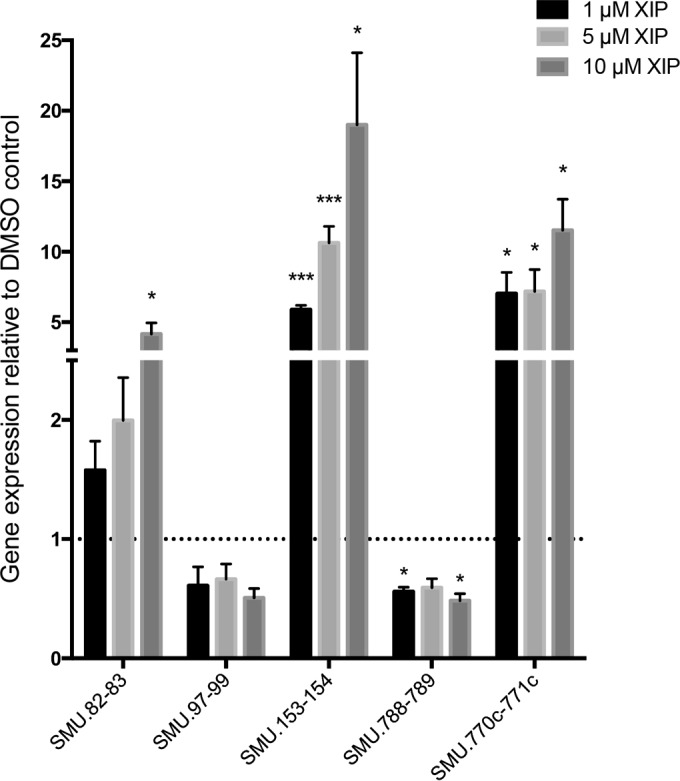
Expression of selected intergenic regions in response to increasing concentrations of XIP. qRT-PCR analysis of gene expression was performed with strain UA159 grown in CDM to an OD_600 _of 0.4 in the presence of increasing amounts of XIP. *, *P* < 0.05; ***, *P* < 0.001.

**FIG 6:**
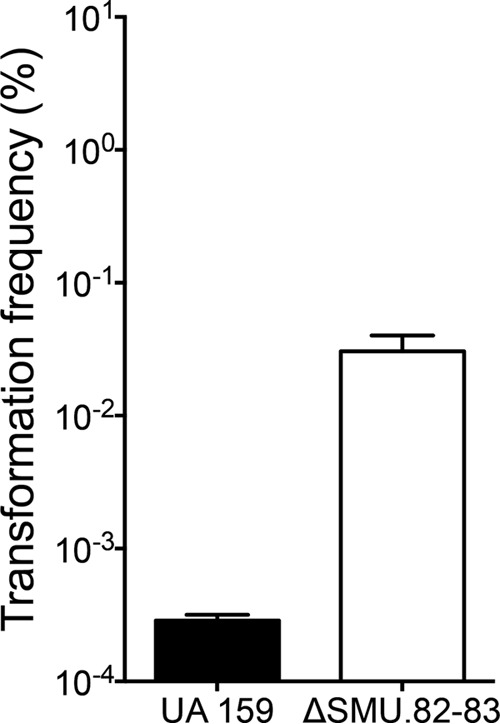
Effect of ΔSMU.82-83 on transformation frequency. Overnight cultures of *S. mutans* UA159 and ΔSMU.82-83 strains were diluted 1:20 in fresh THYE and incubated in the presence or absence of pDL277 (Spec^r^). The resulting percent transformation frequencies are shown. Values are means plus standard errors (error bars) from three independent experiments.

Intergenic regions serve several important regulatory functions and often contain small RNAs (sRNAs). These small transcripts can bind to proteins to regulate their function or base pair with RNAs, modulating their stability and translation. Identification and characterization of sRNAs are therefore important for understanding the regulation of bacterial processes. As a result, we examined large intergenic regions of greater than 500 bp for unidentified transcripts. Of these regions, products were identified in 11 regions, 5 of which were responsive to XIP treatment in *S. mutans* UA159 (Table 1). The transcriptional 5′ start sites were mapped for several of the transcripts and are listed in Table 1. Of note is the intergenic region between SMU.153 and SMU.154, which is located downstream of bacteriocin genes and is induced 5-, 10-, and 20-fold in response to 1 µM, 5 µM, and 10 µM XIP, respectively (Fig. 5). Furthermore, induction of the intergenic region between SMU.82 (*dnaK*) and SMU.83 (*dnaJ*) was observed at higher XIP concentrations. Specifically, this region increased by two- and four-fold in response to 5 µM and 10 µM XIP, respectively. The SMU.82-83 intergenic region is located within the heat shock operon, and in addition to increased transcription with increasing concentrations of XIP, this region has been shown to be upregulated in response to heat stress when mid-logarithmic cells were exposed to high temperatures of 50°C for 20 min (25). A strain with deletion of the intergenic region (ΔSMU.82-83) exhibited increased natural competence compared to strain UA159 (Fig. 6), suggesting a role in negative regulation of competence.

**TABLE 1:** Intergenic regions up- or downregulated in *S. mutans* UA159 in response to 1 µM XIP^a^

Upstream gene	US genedirection	Downstream gene	DS gene direction	sRNA direction	Detected by Northern blotting	5′ RACE start site	XIP sensitivity
SMU.60	>	SMU.61	>	→	Yes	ND	ND
SMU.82	>	SMU.83	>	→	Yes	87,809	Upregulated
SMU.97	>	SMU.99	>	→	Yes	99,434	Downregulated
SMU.97	>	SMU.99	>	←	Yes	99,836	
SMU.97	>	SMU.99	>	←	Yes	99,989	
SMU.97	>	SMU.99	>	→	Yes	99,930	
SMU153	>	SMU.154	>		Yes	ND	Upregulated
SMU.217c	<	SMU.218	>		Yes	ND	ND
SMU.259	>	SMU.260	>		Yes	ND	ND
SMU.305	>	SMU.307	>		Yes	ND	ND
SMU.770c	<	SMU.771c	<	←	No	719,052	Upregulated
SMU.788	>	SMU.789	>		Yes	ND	Downregulated
SMU.1063	<	SMU.1064c	<	←	No	1,008,320	ND
SMU.1405c	<	SMU.1406c	<		Yes	ND	ND

a US, upstream; DS, downstream; ND, not determined.

## DISCUSSION

A microbial population that is genetically competent is better equipped to withstand environmental changes and stressful conditions. The state of competence increases genetic variability, thereby increasing the likelihood of survival for some members of the population during an environmental perturbation (2). Furthermore, access to eDNA provides the competent population with a source of DNA for genetic repair or nutrients during starvation (2). In *S. mutans*, the state of competence is regulated by the competence peptide XIP (6, 7). Since its discovery and implication in genetic transformation, XIP signaling has also been shown to regulate cell lysis and bacteriocin expression (8, 10, 12). Our study further examined the effects of XIP on the transcriptome of *S. mutans* using RNAseq. The cells were exposed to XIP during growth to mid-logarithmic phase to ensure sufficient exposure time for XIP to capture both direct and indirect effects of XIP on gene expression in *S. mutans*. This allowed for a global overview of the transcriptional changes associated with XIP signaling. Additionally, to ensure that any endogenous XIP production in mid-logarithmic WT cells did not mask any effects on the *S. mutans* transcriptome, we also examined the transcriptome of the *comS* deletion strain, which does not produce any endogenous XIP.

**FIG 7:**
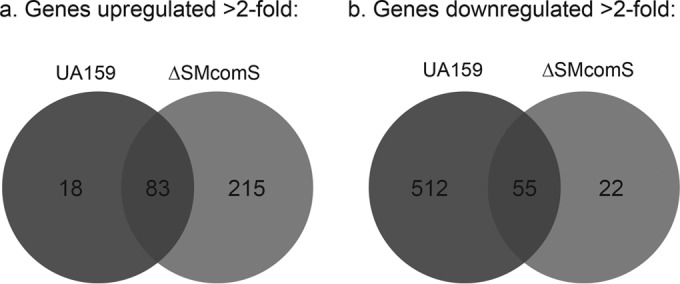
Overlap of the XIP transcriptomes in *S. mutans* UA159 and ΔSMcomS strains. A Venn diagram summarizing the overlap between upregulated (a) or downregulated (b) genes in UA159 and ΔSMcomS strains.

As expected, exposure of both *S. mutans* UA159 and ΔSMcomS with 1 µM XIP resulted in the upregulation of genes involved in cell competence, lysis, and bacteriocin production (6, 8, 10, 26). XIP treatment of strain ΔSMcomS resulted in the strongest upregulation of competence and DNA uptake loci (a transcriptional profile that matches competence induction), while the same treatment of the wild-type strain resulted in the highest increase in genes associated with bacteriocin expression and cell lysis (matching the lytic phenotype previously observed with larger amounts of XIP [8]) (Fig. 1 and Fig. 3). Further differences between the *S. mutans* UA159 and ΔSMcomS XIP transcriptomes were observed in genes outside the core competence and lysis regulons (Fig. 7): while the ΔSMcomS transcriptome in response to 1 µM XIP contained increased expression in genes involved in mobile and extrachromosomal element functions, transport, and transcriptional regulation, many of these genes were downregulated in strain UA159. The wild-type strain also exhibited strong downregulation in loci involved in amino acid biosynthesis, tRNA, protein synthesis, and purines, pyrimidines, and nucleosides. However, in the ΔSMcomS strain, the number of downregulated genes involved in amino acid and purine/pyrimidine biosynthesis was much smaller. In addition, a large number of genes grouped in the protein synthesis and protein fate category were upregulated in the ΔSMcomS mutant. The differences observed in the two strains may be due to the amplification of the XIP signal in the wild-type UA159 strain, which contains an undisrupted ComRS autoregulatory loop. The amplified XIP signal in the wild-type strain may favor expression of genes involved in bacteriocin production and the stringent response and is consistent with the slow growth and increased lytic phenotype we have previously observed with higher concentrations of XIP (8). It is also possible that the full-length ComS prepeptide contains activity distinct from its role as a prepeptide for XIP, and therefore may contribute to the differences in expression profiles observed between UA159 and ΔSMcomS transcriptomes. The differences between the two transcriptomes warrant further investigation in the future.

Overall, our results demonstrated that XIP induces a global change in gene expression in *S. mutans* that supports not only DNA uptake, repair mechanisms, and bacteriocin production but also induces a global change in expression that favors genes involved in stress adaptation and virulence. We show that in addition to *comCDE*, 1 µM XIP also induced the transcription of *hdrRM*, *brsRM*, and *vicRKX* signaling systems (Fig. 4). All of these systems have previously been implicated in one or more of the phenotypes associated with CSP signaling, including genetic transformation and bacteriocin production (13, 14). The *vicRKX* operon is also an important regulator of acid tolerance, bacterial adherence, and biofilm formation (16–19).

XIP treatment also resulted in upregulation of the *relQ* (p)ppGpp synthase and repression of genes involved in cell growth and replication, a transcriptional profile that is consistent with induction of the stringent response, usually activated in response to starvation (Fig. 1 and Fig. 3). Collectively referred to as (p)ppGpp, this group of signaling molecules includes guanosine 3′-diphosphate, GTP, and guanosine 3′,5′-bispyrophosphate, and these molecules are responsible for global transcriptional changes that favor adaptation to a semidormant state, reducing chromosomal replication and growth of the population (23). Furthermore, activation of the stringent response often results in the release of carbon catabolite repression (CCR) (27). CCR mechanisms repress genes involved in the uptake and metabolism of “nonpreferred” sugars to ensure the efficient uptake and utilization of the “preferred” carbohydrates (27). However, during starvation, the transport systems for nonpreferred sugars are derepressed. Similarly to the stringent response, XIP treatment resulted in the downregulation of the ManL regulator of CCR that represses expression of the *lev* operons involved in fructose uptake and metabolism. As expected, a decrease in *manL*, along with an increase in the *levQRST* four-component signaling system that, in response to fructose levels, regulates LevDEFG fructose/mannose phosphotransferase system, resulted in a significant induction of the *levDEFG* operon (Fig. 2) (19, 21, 22, 28). Our transcriptome results therefore suggest that XIP signaling induces a response that mirrors the stringent response to starvation.

In the Gram-positive *Bacillus subtilis*, genetic competence is tightly controlled and coordinated with the production of the (p)ppGpp alarmone and the stringent response (29, 30). This link between genome replication and natural transformation may provide a mechanism that ensures correct incorporation of the transformed DNA into the genome. Although it has previously been shown that the stringent response and genetic competence are coregulated in *S. mutans* (31), this is the first report that indicates the competence regulatory system ComRS may also regulate (p)ppGpp production. The effect of XIP on the accumulation of the (p)ppGpp messengers remains to be examined in future studies.

Our transcriptome data revealed five novel intergenic regions affected by XIP signaling (Fig. 5). Of these five regions, the SMU.153-154 region was strongly upregulated by increasing concentrations of XIP and is located 3′ of bacteriocin synthetic genes, and therefore may be involved in bacteriocin induction. We further investigated the intergenic region SMU.82-83 located within a heat shock operon. This region was previously shown to be induced by heat stress (25), and our transformation assays reveal that it may play a role in competence shutoff (Fig. 6). The mechanism of competence regulation by SMU.82-83 remains to be examined in future experiments.

### Conclusions.

Our work demonstrates that in *S. mutans*, the competence-inducing XIP peptide not only controls DNA transformation and bacteriocin production but also induces a response that resembles the stringent response to amino acid starvation. Further, we report five XIP-responsive intergenic regions expressing putative short RNAs (sRNAs) and provide the first evidence for a heat shock-regulated intergenic region that negatively regulates genetic competence. Furthermore, this work provides a greater understanding of how global gene expression patterns change in response to the peptide XIP. Collectively, these results provide further evidence that multiple stress response mechanisms are linked through the genetic competence signaling pathway in *S. mutans*.

## MATERIALS AND METHODS

### Strains and growth conditions.

*S. mutans* UA159 (32), the ΔSMU.82-83 intergenic deletion strain, and the *comS* deletion strain (ΔSMcomS) (8) were used in this study. The intergenic region was deleted using PCR ligation mutagenesis as described previously (33). Briefly, flanking regions were amplified using primers 82P1f-P2r and 82P3f-P4r (82P1f, 5′-TTCAAGGTGGTGTTATCACTGG-3′; 82P2r, 5′-GGCGCGCCCTCTCCTGATTTTCAAGCTCG-3′; 82P3f, 5′-GGCCGG CCGATTTCTTATTTTTCTTTGAG-3′; 82P4r; 5′-TTCCCCTTGTCCTGCCAACCG-3′), digested, and ligated to an erythromycin resistance (Erm^r^) cassette. The ligated product was used directly for transformation of *S. mutans* UA159 with the aid of synthetic CSP. Following double-crossover homologous recombination, the intergenic region was replaced by an Erm^r^ cassette, and the deletion strain was selected on THYE (Todd-Hewitt broth containing 0.3% yeast extract) plates containing 10 µg/ml erythromycin. The proper insertion of the Erm^r^ cassette was confirmed by PCR and sequencing.

*S. mutans* strains were grown at 37°C with 5% CO_2_ in either Todd-Hewitt broth (Becton, Dickinson, MD) containing 0.3% yeast extract (THYE) or chemically defined medium (CDM). Erythromycin was used as needed at a concentration of 10 µg/ml. Synthetic XIP peptide was synthesized using 9-fluorenylmethoxy carbonyl (F-MOC) chemistry (Advanced Protein Technology Centre, Hospital for Sick Kids, Toronto, Ontario, Canada). Stock concentrations of 1 µM XIP were prepared in dimethyl sulfoxide (DMSO).

### RNA extraction and preparation for sequencing.

Bacterial cultures of *S. mutans* UA159 and ΔSMcomS used for RNA extraction were grown overnight in THYE, washed, and resuspended in fresh CDM. The cultures were then diluted 1:20 and grown to an optical density at 600 nm (OD_600_) of 0.4 to 0.5 in the presence of 1 µM XIP or 1% DMSO vehicle control. Cells were harvested by centrifugation and resuspended in Trizol reagent (Invitrogen) prior to RNA isolation using the FastPrep system (Bio 101 Savant) as specified by the manufacturer. Total RNA was purified using the RNeasy purification kit and enriched for mRNA with the MicrobExpress kit to remove 23S and 16S rRNA.

### Preparation of mRNA libraries for Illumina deep sequencing (RNAseq).

mRNA was fragmented into 50- to 200-nucleotide fragments by incubation in RNA fragmentation reagent (Ambion) for 15 min. Strand-specific cDNA libraries were prepared by the method of Parkhomchuk et al. (34). Briefly, the fragmented mRNA was then converted into double-stranded cDNA using the SuperScript double-Stranded cDNA synthesis kit (Invitrogen), per the manufacturer’s instructions with the following changes. After the first strand synthesis, the products were purified using Illustra Microspin G-50 columns to remove all the deoxynucleoside triphosphates (dNTPs), and a dUTP mixture containing dUTP, in lieu of dTTP, was used for the second strand synthesis. Samples were sequenced by the University of Vermont Cancer Center Advanced Genome Technologies Core. Each sample was sequenced at a minimum of 1,000× coverage using 100-bp single-end reads on an Illumina HiSeq1000 instrument. *Staphylococcus aureus* was included as a control for strandedness.

### Data analysis.

RNAseq data were analyzed using the Galaxy server hosted by the research computing center at the University of Florida by the method of Zeng and Burne (35). Briefly, reads were mapped to the *S. mutans* UA159 genome using Map with Bowtie for Illumina (version 1.1.2), with the default setting for single-end reads. Mapped reads corresponding to each gene were counted using htseq-count for both sense and antisense transcripts. The data presented in Table 1 are based on standardized mapped reads for the respective genes from single biological samples. Count data were standardized by reads per kilobase per million total reads per sample (RPKM). Specific results of interest were verified by quantitative real-time PCR (qRT-PCR).

### qRT-PCR.

Overnight cultures of wild-type UA159 and ΔSMcomS strain in THYE were washed and resuspended in CDM. The cultures were then further diluted 20 times in fresh CDM and grown to an OD_600_ of 0.4 to 0.5 in the presence of different concentrations of XIP. RNA isolation, DNase treatment, cDNA synthesis, qRT-PCR, and expression analyses were carried out as previously described (8). Primers used for qRT-PCR can be found in Table S1 in the supplemental material. Expression was normalized to that of the 16S rRNA gene, and statistical analyses were performed on four independent experiments using Student’s *t* test (*P* < 0.05).

10.1128/mSystems.00102-16.1 TABLE S1  qRT-PCR primers. Download TABLE S1, PDF file, 0.05 MB. Copyright © 2017 Wenderska et al. 2017 Wenderska et al. This content is distributed under the terms of the Creative Commons Attribution 4.0 International license.

10.1128/mSystems.00102-16.2 TABLE S2  Genes up- or downregulated in *S. mutans* UA159 in response to 1 µM XIP. Download TABLE S2, PDF file, 0.2 MB. Copyright © 2017 Wenderska et al. 2017 Wenderska et al. This content is distributed under the terms of the Creative Commons Attribution 4.0 International license.

10.1128/mSystems.00102-16.3 TABLE S3  Genes up- or downregulated in *S. mutans* ΔSMcomS in response to 1 µM XIP. Download TABLE S3, PDF file, 0.1 MB. Copyright © 2017 Wenderska et al. 2017 Wenderska et al. This content is distributed under the terms of the Creative Commons Attribution 4.0 International license.

### Northern blot detection.

Total RNA was isolated from *S. mutans* UA159 cultures using Direct-zol RNA miniprep (Zymo Research). Five micrograms of total RNA was loaded into each lane and resolved on an 8% (wt/vol) polyacrylamide denaturing gel with 7.0 M urea. The RNA was transferred to a nylon membrane (Fermentas) and cross-linked using UV light for 5 min. The membranes were prehybridized in digoxigenin (DIG) Easy Hyb buffer (Roche) for 30 min at 42°C which was followed by a hybridization step using DIG High Prime DNA probes (25 ng/ml) in DIG Easy Hyb buffer at 42°C overnight. Genomic DNA was amplified using region-specific primers to generate probes that were then labeled using the DIG High Prime Labeling kit (Roche) following the supplier’s instructions. The blot was stringently rinsed in 2× SSC (1× SSC is 0.15 M NaCl plus 0.015 M sodium citrate) and again in 0.5× SSC at 60°C. The remaining steps were performed at room temperature. The blot was washed with 0.1 M maleic acid buffer (MAB), blocked with 1× blocking solution (Roche) in 0.1 M MAB, and probed with DIG antibody (75 mU/ml) diluted 1:10,000 in blocking solution. The membrane was then incubated with disodium 3-(4-methoxyspiro {1,2-dioxetane-3,2'-(5'-chloro)tricyclo [3.3.1.13,7]decan}-4-yl)phenyl phosphate (CSPD) (Roche), which resulted in a chemiluminescent signal that was visualized using a chemiluminescent detector (Bio-Rad). The transcript expression levels were quantified by densitometry using the ImageJ64 program (National Institutes of Health, Bethesda, MD). The 5S rRNA was used as a loading control.

### Rapid amplification of cDNA ends.

The transcription start and stop sites were mapped for several intergenic regions. The start sites were determined by using 5′ rapid amplification of cDNA ends (RACE) techniques using the RNA ligase-mediated RACE (RLM-RACE) kit (Thermo Fisher, MA). Briefly, 10 μg of total RNA was treated using calf intestinal phosphatase. The RNA was then treated using tobacco acid pyrophosphatase and ligated to an adapter sequence (5′-GCUGAU GGCGAUGAAUGAACACUGCGUUUGCUGGCUUUGAUGAAA-3′), which was reverse transcribed using an outer primer (5′-TCACAGAGTCGTAGGCGTAT-3′). PCR was then performed using an inner nested primer with a region-specific primer to amplify the region along the transcript; this DNA was then pooled, column purified, and sequenced by ACGT (Toronto, Ontario, Canada). The sequenced result was aligned to the NCBI sequence of the same region in order to locate the 5′ end.

A search for 3′ stop sites was also performed. The procedure used the RLM-RACE kit (Thermo Fisher, MA) according to the manufacturer’s instructions. Briefly, 1 μg of total RNA was reverse transcribed in the presence of the 3′ RACE adapter (5′-GCGAGCACAGAATTAATACGACTCACTATAGGT12VN-3′). The reaction was then PCR amplified using the 3′ RACE adapter primer and a region specific primer (5′-CGAAAATCTCACGTCATGAC-3′). To determine the location of the 3′ end, the resulting PCR product was column purified, sequenced, and aligned to the NCBI sequence of the same region.

### Transformation assay.

To perform genetic transformation assays, an overnight culture of *S. mutans* grown in THYE at 37°C was centrifuged at 4,500 rpm for 4 min at room temperature and resuspended in 1 volume of THYE. The washed culture was diluted 1:20 in THYE. Aliquots (200 μl) of the culture were transferred into a sterile microplate (Costar; Corning, Corning, NY). In test samples, 1 μg of plasmid (pDL277) was added. The sample was incubated at 37°C with 5% CO_2_ under static conditions. After 5 h, test samples (20 μl using serial dilutions in phosphate-buffered saline [PBS]) with or without plasmid were plated on THYE plates supplemented with spectinomycin (10 μg/ml). After 48 h of incubation at 37°C, the transformation frequency was calculated as percent survivability by counting the number of antibiotic-resistant CFUs, which was then divided by the total number of viable CFUs × 100. The assay was performed with a minimum of three biological replicates and three technical replicates.
