# Supplementary material for: Transcriptional Profiling of the Oral Pathogen Streptococcus mutans in Response to Competence Signaling Peptide XIP
Source: mSystems. 2017 Jan 3;2(1):e00102-16. doi: 10.1128/mSystems.00102-16 (PMC5209530; doi:10.1128/mSystems.00102-16)
Supplement: TABLE S1 [file sys001172075st1.pdf]

Table S1. qRT-PCR primers

Primer Name	Primer Sequence
comC-for	GACTGATGAATTAGAGATTATCATTGG
comC-rev	TTTCCCAAAGCTTGTGTA AAACT
comD-for	CGATGCTGTCAAGGGTATCTTGTC
comD-rev	CAAGCAACTCCATCTCAGGCAG
comE-for	CCTGAAAAGGGCAATCACCAG
comE-rev	GGGGCATAAACTCAGAATGTG TCG
relQ-for	CGGTAAAGTTCATCTGTGTCATCAC
relQ-rev	AGACGAGGAAATGCGGCAG
vicR-for	CGCAGTGGCTGAGGAAAATG
vicR-rev	ACCTGTGTGTGTCGCTAAGTGATG
vicK-for	CGCAGTGGCTGAGGAAAATG
vicK-rev	ACCTGTGTGTGTCGCTAAGTGATG
vicX-for	TGCTCAACCACAGTTTTACCG
vicX-rev	GGACTCAATCAGATAACCATCAGC
levD-for	GGAAGCCCTTTGACAAC AGC
levD-rev	CTGCCATTGGTAAGTTCATCCC
levR-for	ACATCTGGATTAATCATGGC
levR-rev	AAAGCTCTTCAATATGGTGC
manL-for	TGGCTATCGGAATCGTTATCGC
manL-rev	ATCATCAGGTCCTTCACTTGGC
hdrR-for	AAGCCATTTGCTTCTGCG
hdrR-rev	TGGGGGTAGAGGAGAAAGAC
hdrM-for	GGTGAAACCAATCTGCGTATTC
hdrM-rev	CCATTTGTGCTAGGAAAACCTG
brsR-for	CACGGAAAACAAACAGGTC
brsR-rev	TTCACCTTGGGAGATACG
brsM-for	GGCGTTTTACAAGGATTTGC
brsM-rev	GCTAAGAGAAGTGGTAGGACAATG
SMU.82-83-for	ACTAAACACAGGCGTTAGG
SMU.82-83-rev	CCAGGGTATCTAGGAGGTATT
SMU.97-99-for	ACAAAGCAATCAGCATCCAAAG
SMU.97-99-rev	CGTTTTGTTGGCAGCCAATTTC
SMU.153-154-for	CAATCTGTACTGATCCCC
SMU.153-154-rev	AAACCATCCCTCTCTAATTCA
SMU.770c-771c-for	CAGAGATGTCTAAACAATCAGA
SMU.770c-771c-rev	ACATGCTCCTTTTAGCATACA
SMU.788-799-for	GCTTTGACTTTCGCTACATT
SMU.788-799-rev	ACAGACTAATCACTCCGC
