# Supplementary material for: Transcriptional Profiling of the Oral Pathogen Streptococcus mutans in Response to Competence Signaling Peptide XIP
Source: mSystems. 2017 Jan 3;2(1):e00102-16. doi: 10.1128/mSystems.00102-16 (PMC5209530; doi:10.1128/mSystems.00102-16)
Supplement: TABLE S2 [file sys001172075st2.pdf]

Table S2. Genes up- or down-regulated in *S. mutans* UA159 in response to 1 μM XIP.

Gene ID	Fold Change	Annotation
**Cell killing and bacteriocin production:**		
SMU.1910c	26.0	hypothetical protein
SMU.1912c	24.2	hypothetical protein
SMU.1913c	19.0	putative immunity protein; BlpL-like; immA
SMU.1914c	31.9	hypothetical protein; nlmC; mutacin V
SMU.1908c	22.1	hypothetical protein; immunity protein
SMU.1909c	21.7	hypothetical protein
SMU.1903c	20.0	putative bacteriocin secretion protein; bsmL
SMU.1904c	18.3	hypothetical protein
SMU.1905c	18.4	hypothetical protein
SMU.1906c	17.9	bacteriocin-related protein
SMU.150	10.4	nlmA; non-lantibiotic mutacin IV A
SMU.151	14.0	nlmB; non-lantibiotic mutacin IV B
SMU.152	8.9	hypothetical protein
SMU.153	7.8	hypothetical protein
SMU.423	10.2	nlmD; possible bacteriocin
SMU.836	8.9	hypothetical protein
SMU.925	2.5	cipI; bacteriocin immunity protein
SMU.1917	3.6	comE; response regulator
SMU.1916	2.8	comD; histidine kinase
SMU.1915	2.9	comC; competence stimulating peptide
SMU.168	2.8	transcriptional regulator toxin-antitoxin
SMU.166	2.8	putative toxin-antitoxin system
SMU.167	3.1	putative toxin-antitoxin system
SMU.2035	-2.0	possible bacteriocin self-immunity protein
**Competence and DNA transformation:**		
SMU.2086	4.5	competence damage-inducible protein A
SMU.1983	5.6	competence protein comYD
SMU.1984	5.5	competence protein comYC
SMU.1981c	5.3	competence protein comG
SMU.1001	5.2	dprA; DNA processing protein; Smf family
SMU.1997	4.4	comX; competence-specific sigma factor
SMU.625	3.9	comEA; competence protein
SMU.626	3.8	comEC; competence protein
SMU.498	3.4	comFA; late competence protein F
SMU.499	4.0	comFC; late competence protein
SMU.644	2.0	coiA; competence protein

SMU.1981c	5.3	competence protein G
SMU.1985	5.5	comGB; competence protein
SMU.1987	7.6	comGA; late competence protein
SMU.1881c	-2.4	competence factor transporting ATP-binding / permease protein
**Mobile and extrachromosomal element functions:**		
SMU.1817c	-3.7	transposon-related, maturase-related protein fragment
SMU.1816c	-3.2	transposon-related, maturase-related protein fragment
SMU.226c	-2.6	transposase, IS1216, also reported on plasmid, truncated
SMU.106c	-2.5	transposase fragment
SMU.1332c	-2.3	transposase
SMU.1374	-2.1	IS30 transposase related protein
SMU.1329c	-2.1	transposase fragment
SMU.1331c	-2.1	transposase
SMU.1355c	-2.1	transposase
SMU.1330c	-2.0	transposase, IS1167, fragment
SMU.94c	3.3	transposase fragment
SMU.1024c	-3.8	transposase fragment
SMU.1029	-9.2	hypothetical – transposon
SMU.93c	-2.1	putative transposase
SMU.1407c	-2.0	transposase, ISSmu1
**Pathogenesis and toxin production:**		
SMU.610	-3.5	cell surface antigen
SMU.1396	-2.4	glucan-binding protein C
**Cell division:**		
SMU.454	-2.1	cell division protein
SMU.1394	-2.0	GTP-binding protein LepA
SMU.1276c	-2.0	cell division regulator
SMU.551	-2.0	cell division protein FtsA
SMU.1003	3.5	glucose-inhibited division protein
**DNA metabolism, replication, recombination and repair:**		
SMU.1055	8.2	DNA repair protein RadC
SMU.1967	7.9	ssbA; single-stranded DNA-binding protein
SMU.505	7.5	dpn; adenine-specific DNA methylase
SMU.506	5.8	ssuRB; type II restriction endonuclease
SMU.1002	3.6	topA; DNA topoisomerase I
SMU.2085	3.4	recA; recombinase A

SMU.64	2.7	ruvB; holliday junction DNA helicase
SMU.60	2.1	alkD; DNA alkylation repair enzyme
SMU.510c	-3.3	deoxyribonuclease
SMU.1258c	-2.6	type II restriction endonuclease subunit
SMU.342	-2.5	possible primase-related protein
SMU.1313c	-2.4	ATP-dependent DNA helicase
SMU.469	-2.3	recombination protein U
SMU.1859	-2.2	single-stranded DNA-binding protein
SMU.1114	-2.1	DNA gyrase A subunit
SMU.1650	-2.1	endonuclease III (DNA repair)
SMU.1192	-2.0	DNA-polymerase III subunit alpha
SMU.1714c	-2.0	tyrosine recombinase
SMU.2088	-2.0	Holliday junction DNA helicase
SMU.1581	-2.0	DNA polymerase III subunits gamma and tau
**Transport and binding:**		
SMU.772	3.2	gbpD; bifunctional glucan-binding protein D and lipase
SMU.1966c	2.7	levT; ABC transport ribose-binding protein
SMU.651c	2.2	ABC transporter; periplasmic substrate-binding protein
SMU.1963c	2.3	levQ; sugar-binding periplasmic protein
SMU.864	5.6	ABC transporter; permease component
SMU.1195	2.9	ABC transporter permease protein
SMU.863	4.5	ABC transporter, ATP-binding protein
SMU.1194	2.7	yurY; ABC transporter, ATP-binding protein
SMU.1063	-5.0	amino acid ABC transporter, ATP-binding protein
SMU.819	-2.0	large conductance mechanosensitive channel
SMU.770c	4.8	manganese transporter/possible HitA ferric iron-binding periplasmic protein
SMU.1175	-2.5	dagA; sodium:alanine (or glycine) symporter
SMU.432	-3.3	cylB; ABC transporter, permease and solute binding protein
SMU.1062	-5.0	glycine-betaine binding ABC transporter permease
SMU.602	-2.5	sodium-dependent transporter
SMU.408	-2.8	xanthine/uracil permease family protein
SMU.1289c	-2.7	voltage-gated chloride channel family
SMU.819	-2.0	large conductance mechanosensitive channel
SMU.1176	-2.3	cation efflux protein
SMU.1852	-2.3	cation transporter, CorA family
SMU.121	-2.3	MATE efflux family , DinF
SMU.540	-2.2	peroxide resistance protein / iron binding protein
SMU.723	-2.2	calcium-transporting ATPase
SMU.71	-2.4	MATE efflux family protein

SMU.872	-2.0	fructose-specific PTS system enzyme IIBC component
SMU.396	-2.9	glycerol uptake facilitator protein
SMU.1137	-2.2	phosphate ABC transporter, permease
SMU.388	-2.2	possible branched-chain amino acid permease
SMU.951	-2.1	amino acid permease
SMU.1095	-2.4	proline/glycine betaine ABC permease and solute binding protein
SMU.1669	-2.4	branched-chain amino acid ABC transporter, substrate-binding protein
SMU.1121c	-2.4	ribonucleoside ABC transporter, solute-binding protein
SMU.2118	-2.1	glycine betaine/carnitine/choline ABC transporter, substrate-binding protein
SMU.1177c	-2.0	amino acid ABC transporter, amino acid-binding protein
SMU.1447c	-2.0	ABC transporter, substrate-binding protein
SMU.817	-2.0	amino acid ABC transporter, substrate-binding protein
SMU.1136	-2.0	phosphate ABC transporter, permease
SMU.242c	-2.6	glutamine ABC transporter, solute binding protein
SMU.1164c	-2.9	ABC transporter, ATPase component
SMU.1810	-2.6	ABC transporter, membrane spanning permease
SMU.922	-2.6	ABC-type multidrug / protein/ lipid transport system, ATPase component
SMU.1550c	-2.6	ABC transporter, membrane spanning permease
SMU.1668	-2.5	branched chain amino acid ABC transporter, permease
SMU.567	-2.5	amino acid (glutamine) ABC transporter permease
SMU.1119c	-2.4	ribonucleoside ABC transporter permease
SMU.1163c	-2.4	ABC transporter, ATPase component
SMU.2117	-2.4	glycine betaine / carnitine / choline ABC transporter permease
SMU.1166c	-2.4	ABC transporter permease
SMU.1667	-2.4	branched chain amino acid ABC transporter, permease
SMU.1118c	-2.4	ribonucleoside ABC transporter permease
SMU.257	-2.3	oligopeptide ABC transporter, permease
SMU.256	-2.3	oligopeptide ABC transporter, permease
SMU.1216c	-2.3	ABC transporter, amino acid permease
SMU.996	-2.3	ABC transporter, permease protein;possible ferrichrome transport system
SMU.656	-2.1	ABC transporter, permease, possibly bacteriocin associated
SMU.1179c	-2.0	amino acid ABC transporter, permease
SMU.1446c	-2.0	ABC transporter, permease
SMU.2148c	-2.0	ABC transporter, membrane-spanning permease
SMU.1570	-2.0	maltose / maltodextrin ABC transport system (permease)

SMU.1569	-2.0	maltodextrin ABC transport system permease
SMU.923	-2.3	ABC-type multidrug/protein/lipid transport system,ATPase component
SMU.1063	-4.9	amino acid ABC transporter, ATP-binding protein
SMU.1315c	-2.6	ABC transporter, ATP-binding protein
SMU.1695	-2.5	ABC transporter, ATP-binding protein; possible molybdenum transport system
SMU.1178c	-2.5	amino acid ABC transporter, ATP-binding protein
SMU.1096	-2.5	ABC transporter, ATP-binding protein
SMU.997	-2.4	inorganic ion ABC transporter,ATP-binding protein; possible ferrichrome transport system
SMU.1666	-2.4	branched chain amino acid ABC transporter, ATP-binding protein
SMU.1120	-2.3	ribonucleoside ABC transporter, ATP-binding protein
SMU.241c	-2.3	amino acid ABC transporter, ATP-binding protein
SMU.1551c	-2.3	ABC transporter, ATP-binding protein
SMU.2116	-2.3	glycine betaine / carnitine / choline ABC transporter, ATP-binding protein, opuCA
SMU.568	-2.2	glutamine ABC transporter, ATP-binding protein
SMU.1431c	-2.1	ABC transporter, ATP-binding protein
SMU.1519	-2.1	glutamine ABC transport, ATP-binding protein
SMU.1571	-2.1	ABC-type transport system ATP-binding protein (maltose)
SMU.1006	-2.0	ABC transporter, ATP-binding protein
SMU.2149c	-2.0	ABC transporter, ATP-binding protein
SMU.1445c	-2.0	ABC transporter, ATP-binding protein
SMU.1665	-2.0	branched chain amino acid ABC transporter, ATP-binding protein
SMU.1167c	-2.0	ABC transporter ATP-binding protein
SMU.258	-2.0	oligopeptide ABC transporter, ATP-binding
SMU.1811	-2.0	cdd4-like bacteriocin component, ScnF homolog
SMU.2006	-2.3	preprotein translocase
SMU.1348c	-2.2	ABC transporter ATP-binding protein
SMU.1365c	-2.0	Permease-FtsX-like permease
SMU.1366c	-2.0	ABC transporter ATP-binding Protein
**Signal transduction and transcriptional regulation:**		
SMU.507	4.2	transcriptional regulator, DeoR family
SMU.1193	3.9	transcriptional regulator, GntR family
SMU.168	2.8	transcriptional regulator
SMU.1965c	2.7	levS, histidine kinase
SMU.1964c	2.4	levR, two-component response regulator
SMU.1960c	2.5	levE; fructose-specific Enzyme IIB component

SMU.1957	2.4	levG; fructose-specific Enzyme IID component
SMU.1961c	2.2	levD; fructose-specific Enzyme IIA component
SMU.1958c	2.3	levF; fructose-specific Enzyme IIC component
SMU.2038	-2.4	pttB; phosphotransferase system
SMU.1878	-3.9	manM; mannose PTS system component IIC
SMU.1053	6.0	possible redox-sensing transcriptional repressor Rex
SMU.1877	-3.8	manL; mannose PTS system component IIAB
SMU.1879	-4.2	manD; mannose PTS system component IID
SMU.1064c	-3.0	transcriptional regulator
SMU.1065c	-3.3	transcriptional regulator, GntR family
SMU.1165c	-3.0	transcriptional regulator (TetR/AcrR family)
SMU.1419	-2.7	transcriptional regulator
SMU.921	-2.5	transcriptional regulator
SMU.433	-2.5	transcriptional regulator
SMU.702c	-2.5	transcriptional regulator
SMU.2058	-2.4	transcriptional regulator
SMU.2060	-2.3	transcriptional regulator, LysR family (possible RUBISCO transcriptional regulator, RscR)
SMU.1025	-2.2	transcriptional regulator
SMU.1097c	-2.2	transcriptional regulator
SMU.2108c	-2.1	transcriptional regulator, MepR protein (possible regulator of MepA)
SMU.1745c	-2.1	transcriptional regulator, MarR family
SMU.1168	-2.0	transcriptional regulator (TetR/AcrR family)
SMU.124	-2.0	transcriptional regulator, MarR family
SMU.2001	-2.2	DNA-directed RNA polymerase, alpha subunit
SMU.1990	-2.0	DNA-directed RNA polymerase, beta subunit
SMU.336	-2.1	ribonuclease P protein component
SMU.1607	-2.0	exoribonuclease R
SMU.577	-2.5	sensor histidine kinase
SMU.1547c	-2.4	response regulator
SMU.1548c	-2.4	sensor histidine kinase
SMU.576	-2.3	response regulator
SMU.660	-2.1	histidine kinase
SMU.2038	-2.4	phosphotransferase system, trehalose-specific IIBC component (EIIBC-tre)
SMU.115	-2.1	PTS system, fructose-specific IIA component
SMU.2097	-2.3	arginine transcriptional repressor (arginine hydroxymate resistance protein)
SMU.292	-2.1	transcriptional regulator, probable AraC family
SMU.105	-2.0	SCR operon transcriptional repressor
SMU.2134	-2.1	transcriptional regulator, TetR/AcrR family

SMU.1225	-2.1	transcriptional regulator, LysR family; probable metCcysK operon transcriptional activator
**Fatty acid and phospholipid metabolism:**		
SMU.1743	-2.5	acyl carrier protein
SMU.1785	-2.1	phosphatidate cytidylyltransferase (CDP-diglyceride synthase)
SMU.1744	-2.3	3-oxoacyl-[acyl-carrier-protein] synthase III
SMU.624	-2.2	1-acylglycerol-3-phosphate O-acyltransferase
SMU.1417c	-2.1	acyl-ACP thioesterase
SMU.988	-2.0	cardiolipin synthase
**Energy metabolism:**		
SMU.671	3.3	citrate synthase
SMU.672	2.9	isocitrate dehydrogenase
SMU.670	2.6	aconitate hydratase; aconitase A
SMU.646	2.2	hydrolase (possible phosphoglycolate phosphatase)
SMU.352	2.4	ribulose-phosphate-3-epimerase
SMU.1978	4.9	acetate kinase
SMU.2042	-2.2	dextranase (1,6-alpha-glucanhydrolase)
SMU.2047	-2.1	PTS system, enzyme II, A component
SMU.1496	-2.1	galactose-6-phosphate isomerase
SMU.2065	-2.0	UDP-glucose 4-epimerase
SMU.1278c	-2.0	phosphoglycolate phosphatase
SMU.1123	-2.2	deoxyribose-phosphate aldolase
SMU.291	-2.1	transketolase
SMU.942	-2.0	3-hydroxy-3-methylglutaryl-CoA reductase
SMU.1104c	-2.1	phosphoglycerate mutase-like protein
SMU.1191	-2.5	6-phosphofructokinase
SMU.700c	-2.3	phosphoglycerate mutase
SMU.1190	-2.2	pyruvate kinase
SMU.1010	-2.0	citrate lyase synthetase
SMU.1420	-2.3	NADPH-quinone reductase
SMU.1971c	-2.1	thioredoxin family protein
SMU.1432c	-2.6	glycosyl hydrolases family 8 protein; possible beta-glucanase
SMU.2037	-2.2	trehalose-6-phosphate hydrolase
**Central intermediary metabolism:**		
SMU.1180	-2.2	alkylphosphonate uptake protein
SMU.318	-2.3	peptidase/aminoacylase/hippurate hydrolase
SMU.1595	-2.3	carbonic anhydrase (carbonate dehydratase)

SMU.1635	-2.0	UDP-N-acetylglucosamine pyrophosphorylase
SMU.939	-2.3	isopentenyl-diphosphate delta-isomerase
SMU.943c	-2.0	hydroxymethylglutaryl-CoA synthase
SMU.1322	2.3	acetoin reductase
SMU.663	2.7	N-acetyl-gamma-glutamyl-phosphate reductase (N-acetyl-glutamate-gamma-semialdehyde dehydrogenase)
SMU.1437	-2.3	UDP-N-acetylglucosamine 2-epimerase
**Purines, pyrimidines, nucleosides, and nucleotides:**		
SMU.356	2.1	purine operon repressor/xanthine phosphoribosyltransferase
SMU.1050	-2.0	prs; phosphoribosyl pyrophosphate synthetase
SMU.37	-3.8	purH; phosphoribosylaminoimidazolecarboxamide formyltransferase / IMP cyclohydrolase
SMU.32	-4.6	purB; amidophosphoribosyltransferase
SMU.59	-5.7	asl; adenylosuccinate lyase
SMU.50	-5.6	purE; phosphoribosylaminoimidazole carboxylase catalytic subunit
SMU.34	-5.0	purM; phosphoribosylformylglycinamide cyclo-ligase (AIRS)
SMU.51	-5.8	purK; phosphoribosylaminoimidazole carboxylase, ATPase subunit
SMU.35	-5.2	purN; phosphoribosylglycinamide formyltransferase
SMU.48	-6.5	purD; phosphoribosylamine-glycine ligase
SMU.30	-6.9	purL; phosphoribosylformylglycinamidine synthase
SMU.29	-9.6	purC; phosphoribosylaminoimidazole-succinocarboxamide synthase
SMU.1054	10.1	guaA; glutamine amidotransferase
SMU.595	-2.4	dihydroorotate dehydrogenase (dihydroorotate oxidase)
SMU.1223	-2.1	dihydroorotate dehydrogenase
SMU.1050	-2.0	phosphoribosyl pyrophosphate synthetase
SMU.1066	-2.1	GMP synthase
SMU.2157	-2.1	inosine-5'- monophosphate dehydrogenase
SMU.1122	-2.6	cytidine deaminase
SMU.1215	-2.3	uracil-DNA glycosylase
SMU.2005	-2.1	adenylate kinase (ATP-AMP transphosphorylase) (superoxide-inducible protein 16)
SMU.1386	-2.0	uridine kinase
SMU.668c	-2.6	ribonucleotide reductase, large subunit
SMU.667	-2.2	ribonucleotide reductase, small subunit
SMU.2074	-2.1	anaerobic ribonucleoside-triphosphate reductase
**Amino acid biosynthesis:**		

SMU.54	-6.3	amino acid racemase
SMU.531	-2.4	chorismate mutase; possible prephenate dehydrogenase
SMU.538	2.2	tryptophan synthase, alpha subunit
SMU.1312	-2.3	aspartate aminotransferase
SMU.54	-6.3	amino acid racemase
SMU.70	-2.1	threonine synthase
SMU.989	-2.1	aspartate-semialdehyde dehydrogenase
SMU.449	-2.5	gamma-glutamyl kinase
SMU.450	-2.4	gamma-glutamyl phosphate reductase
SMU.1263	-2.3	phosphoribosyl-AMP cyclohydrolase/phosphoribosyl-ATP pyrophosphatase
SMU.1266	-2.4	amidotransferase; possible imidazoleglycerol-phosphate synthase
SMU.1268	-2.7	imidazoleglycerol-phosphate dehydratase
SMU.1270	-2.4	histidinol dehydrogenase
SMU.1271	-2.9	ATP phosphoribosyltransferase
SMU.1273	-2.3	histidinol-phosphate aminotransferase
SMU.233	-2.4	ketol-acid reductoisomerase
SMU.231	-2.7	acetolactate synthase, large subunit (AHAS)
SMU.232	-2.6	acetolactate synthase, small subunit
SMU.1269	-2.4	phosphoserine phosphatase
SMU.965	-2.0	homoserine dehydrogenase
SMU.966	-2.0	homoserine kinase
SMU.1265	-2.0	phosphoribosylformimino-5-aminoimidazole carboxamide ribotide isomerase
SMU.1264	-2.0	cyclase HisF/ imidazoleglycerol-phosphate synthase
SMU.536	2.0	phosphoribosylanthranilate isomerase
**Biosynthesis of cofactors, prosthetic groups, and carriers:**		
SMU.1827	-3.1	biotin biosynthesis protein
SMU.967	-2.1	bifunctional protein: folylpolyglutamate synthase/dihydrofolate synthase
SMU.970	-2.3	dihydroneopterin aldolase
SMU.838	4.1	glutathione reductase
SMU.2063	-2.2	ferrochelatase (heme synthetase) (protoheme ferro-lyase)
SMU.917c	-6.6	6-pyruvoyl tetrahydropterin synthase, PTPS
SMU.954	-2.1	pyridoxal kinase
SMU.85	-2.5	phosphomethylpyrimidine kinase
**Cell envelope, biosynthesis and degradation of murein sacculus and peptidoglycan:**		
SMU.1572	-2.1	UDP-N-acetylglucosamine 1-carboxyvinyltransferase

SMU.20	-2.4	cell shape-determining protein MreC
SMU.253	-2.1	D-alanyl-D-alanine carboxypeptidase; penicillin-binding protein
SMU.599	-2.1	D-alanine-D-alanine ligase
SMU.707c	-2.9	endolysin
SMU.1786	-2.1	undecaprenyl pyrophosphate synthetase
**Adaptation to atypical conditions and detoxification:**		
SMU.1059	-2.1	acid tolerance protein
SMU.669c	-3.6	glutaredoxin
SMU.919c	-7.6	ATPase, confers aluminum resistance
SMU.1286c	-2.1	multidrug resistance permease
SMU.2109	-2.2	possible multiple drug efflux pump MepA
SMU.745	-2.8	drug-export protein; multidrug resistance protein
SMU.244	-2.7	undecaprenyl-diphosphatase; bacitracin resistance protein
SMU.1057	-2.0	acid tolerance protein
**Protein synthesis and fate:**		
SMU.1477	-2.8	tRNA isopentenylpyrophosphate transferase
SMU.788	-2.6	RNA methyltransferase, TrmA family
SMU.1821c	-2.4	glutamyl-tRNA (Gln) amidotransferase subunit C
SMU.187c	-2.3	probable tRNA-dihydrouridine synthase
SMU.1820c	-2.3	glutamyl-tRNA(Gln) amidotransferase subunit A
SMU.1819	-2.3	glutamyl-tRNA (Gln) amidotransferase subunit B
SMU.868	-2.1	tRNA (guanine-N1)-methyltransferase
SMU.1139c	-2.1	rRNA methyltransferase, NOL1/NOP2/sun family
SMU.1707c	-2.1	spoU-related rRNA/tRNA methylase
SMU.1950	-2.0	ribosomal large subunit pseudouridine synthase D
SMU.1510	-3.2	phenylalanyl-tRNA synthetase, beta subunit
SMU.650	-3.1	alanyl-tRNA synthetase (alanine--tRNA ligase)
SMU.1822	-3.0	aspartyl-tRNA synthetase
SMU.773c	-2.5	lysyl-tRNA synthetase
SMU.558	-2.4	isoleucine-tRNA synthetase
SMU.1272	-2.4	histidyl-tRNA synthetase
SMU.1311	-2.4	asparaginyl-tRNA synthetase
SMU.1512	-2.3	phenylalanyl-tRNA synthetase, alpha subunit
SMU.1992	-2.2	tyrosyl-tRNA synthetase
SMU.1770	-2.2	valyl-tRNA synthetase
SMU.2158c	-2.2	tryptophanyl-tRNA synthetase
SMU.445	-2.2	glycyl-tRNA synthetase alpha subunit
SMU.158	-2.1	cysteinyl-tRNA synthetase

SMU.1943	2.1	leucyl-tRNA synthetase
SMU.608	-2.8	peptide chain release factor 3 ; translation elongation and release factor
SMU.2004	-2.2	translation initiation factor IF-1
SMU.697	-2.1	translation initiation factor IF-3
SMU.1326	-2.1	peptide chain release factor
SMU.2031	-2.0	translation elongation factor Ts
SMU.2022	-2.8	50S ribosomal protein L22
SMU.957	-2.6	50S ribosomal protein L10
SMU.2021	-2.6	30S ribosomal protein S3
SMU.2020	-2.6	50S ribosomal protein L16
SMU.2003	-2.6	30S ribosomal protein S13, N-terminal fragment
SMU.2024c	-2.6	50S ribosomal protein L4, N-terminal fragment
SMU.2025	-2.6	50S ribosomal protein L3
SMU.2002	-2.5	30S ribosomal protein S11
SMU.960	-2.5	50S ribosomal protein L7/L12
SMU.169	-2.5	50S ribosomal protein L13
SMU.2019	-2.4	50s ribosomal protein L29
SMU.2009	-2.4	30S ribosomal protein S5
SMU.2017	-2.4	50S ribosomal protein L14
SMU.2012	-2.4	30S ribosomal protein S8
SMU.2026c	-2.4	30S ribosomal protein S10 fragment
SMU.358	-2.3	30S ribosomal protein S7
SMU.2016	-2.3	50S ribosomal protein L24
SMU.1200	-2.3	30S ribosomal protein S1
SMU.2014	-2.3	30S ribosomal protein S14
SMU.2007	-2.3	50S ribosomal protein L15, N-terminal fragment
SMU.2015	-2.3	50S ribosomal protein L5
SMU.1610	-2.2	50S ribosomal protein L33
SMU.120	-2.2	50S ribosomal protein L28
SMU.2008	-2.2	50S ribosomal protein L30
SMU.849	-2.2	50S ribosomal protein L27
SMU.698	-2.2	50S ribosomal protein L35
SMU.1626	-2.2	50S ribosomal protein L1
SMU.170	-2.2	30S ribosomal protein S9
SMU.2003a	-2.2	50S ribosomal protein L36
SMU.1860	-2.1	30S ribosomal protein S6
SMU.2018	-2.1	30S ribosomal protein S17
SMU.2104a	-2.1	50S ribosomal protein L32
SMU.1288	-2.1	50S ribosomal protein L19
SMU.2023c	-2.1	30S ribosomal protein S19, C-terminal fragment

SMU.1627	-2.0	50S ribosomal protein L11
SMU.357	-2.0	30S ribosomal protein S12
SMU.2011	-2.0	50S ribosomal protein L6 (BL10)
SMU.818	-2.0	30S ribosomal protein S21
SMU.2000	-2.0	50S ribosomal protein L17
SMU.2032	-2.0	30S ribosomal protein S2
SMU.340	-2.0	50S ribosomal protein L34
SMU.500	2.0	ribosome-associated protein
SMU.1051	2.2	aminotransferase (class V); possible iron-sulfur cofactor synthesis protein;pyridoxal-phosphate dependent aminotransferase
SMU.143c	-2.1	polypeptide deformylase (PDF)
SMU.188c	-2.4	Hsp33-like chaperonin
SMU.1787c	-3.1	preprotein translocase, YajC subunit
SMU.1973	-2.5	glutamyl aminopeptidase
SMU.1592	-2.2	proline dipeptidase
SMU.1030	-2.3	polyribonucleotide nucleotidyltransferase
**tRNA:**		
SMU.t01	-2.8	
SMU.t02	-4.6	
SMU.t03	-2.9	
SMU.t04	-2.9	
SMU.t05	-2.1	
SMU.t06	-2.1	
SMU.t08	-2.4	
SMU.t09	-2.9	
SMU.t10	-2.3	
SMU.t11	-2.2	
SMU.t12	-4.1	
SMU.t13	-2.2	
SMU.t14	-2.6	
SMU.t15	-2.5	
SMU.t16	-4.0	
SMU.t17	-3.0	
SMU.t21	-2.4	
SMU.t22	-3.1	
SMU.t23	-2.2	
SMU.t24	-2.4	
SMU.t26	-2.4	
SMU.t27	-3.1	
SMU.t28	-2.3	

SMU.t30	-3.3	
SMU.t31	-2.6	
SMU.t32	-2.5	
SMU.t34	-2.2	
SMU.t36	-2.8	
SMU.t38	-2.6	
SMU.t39	-3.2	
SMU.t41	-3.1	
SMU.t42	-3.4	
SMU.t43	-2.1	
SMU.t44	-2.0	
SMU.t45	-2.4	
SMU.t46	-2.4	
SMU.t47	-2.9	
SMU.t48	-3.1	
SMU.t49	-3.1	
SMU.t50	-2.6	
SMU.t52	-2.3	
SMU.t53	-2.8	
SMU.t55	-2.7	
SMU.t56	-2.6	
SMU.t57	-3.0	
SMU.t58	-2.4	
SMU.t59	-2.2	
SMU.t60	-2.7	
SMU.t61	-3.4	
SMU.t64	-2.7	
SMU_t35	-2.0	
SMU_t40	-2.0	
SMU_t54	-2.0	
**Other:**		
SMU.837	7.2	oxidoreductase, aldo/keto reductase family
SMU.1979c	6.5	ythI ytxK; conserved hypothetical protein, methyltransferase domain
SMU.1982c	6.0	conserved hypothetical protein
SMU.769	5.6	conserved hypothetical protein
SMU.1980c	5.6	conserved hypothetical protein
SMU.663	2.7	argC; N-acetyl-gamma-glutamyl-phosphate reductase (N-acetyl-glutamate-gamma-semialdehyde dehydrogenase)
SMU.508	3.2	conserved hypothetical protein (possible hydrolase)

SMU.847c	-2.4	hypothetical protein
SMU.862	4.1	conserved hypothetical protein
SMU.2076c	2.9	hypothetical protein
SMU.2081	2.3	hypothetical protein
SMU.355	2.5	CMP-binding factor 1
SMU.65	2.4	protein tyrosine-phosphatase
SMU.1322	2.3	acetoin reductase
SMU.1374	-2.1	IS30 transposase related protein
SMU.329	2.2	conserved hypothetical protein
SMU.807	2.2	conserved hypothetical protein (possible membrane protein)
SMU.673	2.1	conserved hypothetical protein
SMU.354	2.0	conserved hypothetical protein
SMU.67	2.0	acyltransferase
SMU.2048	-2.3	hypothetical protein
SMU.1395c	-2.3	hypothetical protein
SMU.222c	-2.3	hypothetical protein (possible integrase fragment)
SMU.223c	-2.6	hypothetical protein
SMU.224c	-2.7	hypothetical protein
SMU.225c	-2.3	hypothetical protein
SMU.277	-4.1	hypothetical protein
SMU.278	-3.7	hypothetical protein
SMU.279	-4.0	hypothetical protein
SMU.281	-4.3	hypothetical protein
SMU.283	-4.9	hypothetical protein
SMU.284	-3.0	hypothetical protein
SMU.285	-4.4	hypothetical protein
SMU.429c	-2.2	hypothetical protein
SMU.600c	-2.9	conserved hypothetical protein
SMU.604	-2.4	conserved hypothetical protein
SMU.621c	-2.3	conserved hypothetical protein
SMU.622c	-2.5	conserved hypothetical protein
SMU.958	-3.3	hypothetical protein
SMU.959c	-2.6	hypothetical protein
SMU.1435c	-2.7	hypothetical protein
SMU.444	-2.5	hypothetical protein
SMU.451	-3.2	hypothetical protein
SMU.2033c	-3.3	conserved hypothetical protein
SMU.709	-3.0	conserved hypothetical protein
SMU.703c	-2.4	conserved hypothetical protein
SMU.706c	-2.1	conserved hypothetical protein

SMU.711	-3.1	conserved hypothetical protein
SMU.649	-3.4	conserved hypothetical protein
SMU.1753c	-3.5	conserved hypothetical protein
SMU.1754c	-4.6	conserved hypothetical protein
SMU.1755c	-4.1	conserved hypothetical protein
SMU.1757c	-4.3	conserved hypothetical protein
SMU.1758c	-4.0	conserved hypothetical protein
SMU.1760c	-4.0	conserved hypothetical protein
SMU.1761c	-4.2	conserved hypothetical protein
SMU.1762c	-3.5	conserved hypothetical protein
SMU.1763c	-3.5	conserved hypothetical protein
SMU.1764c	-3.9	conserved hypothetical protein
SMU.1262c	-2.1	hypothetical protein
SMU.1267c	-2.5	hypothetical protein
SMU.1310	-2.4	hypothetical protein
SMU.1316c	-3.0	hypothetical protein
SMU.1317c	-3.4	hypothetical protein
SMU.940c	-3.5	hemolysin III-related protein
SMU.987	-3.0	cell wall surface anchor family protein
SMU.1750c	-3.7	hypothetical protein
SMU.1752c	-2.6	hypothetical protein
SMU.1628	-2.2	conserved hypothetical protein
SMU.1623c	-2.1	conserved hypothetical protein
SMU.1373c	-2.8	hypothetical protein
SMU.73	-4.0	conserved hypothetical protein
SMU.1896c	-4.1	hypothetical protein
SMU.1895c	-3.5	hypothetical protein
SMU.1861c	-2.0	hypothetical protein
SMU.1794c	-2.1	hypothetical protein
SMU.18	-2.2	hypothetical protein
SMU.1804c	-2.2	hypothetical protein
SMU.1813	-2.9	hypothetical protein, probable transposase fragment
SMU.1818c	-4.3	hypothetical protein
SMU.1000	-4.6	hypothetical protein
SMU.1373c	-2.8	hypothetical protein
SMU.1395c	-2.3	hypothetical protein
SMU.1552c	-2.0	hypothetical protein
SMU.1553c	-2.7	hypothetical protein
SMU.1587c	-2.5	hypothetical protein
SMU.1648c	-2.2	hypothetical protein
SMU.1766c	-2.1	hypothetical protein

SMU.1768c	-2.1	hypothetical protein
SMU.1946	-2.1	conserved hypothetical protein
SMU.2064c	-2.5	hypothetical protein
SMU.2105	-2.1	hypothetical protein
SMU.2106c	-2.1	hypothetical protein
SMU.2111c	-2.2	hypothetical protein
SMU.33	-5.8	hypothetical protein
SMU.344	-2.2	hypothetical protein
SMU.350	-2.4	hypothetical protein
SMU.49	-6.9	hypothetical protein
SMU.501	-4.3	hypothetical protein
SMU.503c	-2.2	hypothetical protein
SMU.545	-2.3	hypothetical protein
SMU.55	-5.1	hypothetical protein
SMU.58	-5.4	hypothetical protein
SMU.594	-2.0	hypothetical protein
SMU.605	-2.3	hypothetical protein
SMU.606	-2.2	hypothetical protein
SMU.620	-2.6	hypothetical protein
SMU.722	-2.6	hypothetical protein
SMU.748	-2.0	hypothetical protein
SMU.771c	4.1	hypothetical protein
SMU.791c	-2.5	hypothetical protein
SMU.999	-2.3	hypothetical protein
SMU.45	-2.0	hypothetical protein
SMU.1399	-2.0	hypothetical protein
SMU.295	-2.0	hypothetical protein
SMU.1771c	-2.0	hypothetical protein
SMU.1907	21.1	hypothetical protein
SMU.1073	-7.5	formate--tetrahydrofolate ligase
SMU.916c	-7.6	conserved hypothetical protein
SMU.914c	-7.5	conserved hypothetical protein
SMU.56	-6.2	Streptococcus-specific protein
SMU.52	-5.8	conserved hypothetical protein
SMU.31	-5.7	conserved hypothetical protein
SMU.53	-5.5	conserved hypothetical protein
SMU.915c	-4.9	conserved hypothetical protein
SMU.941c	-4.8	conserved hypothetical protein
SMU.2146c	-4.6	conserved hypothetical protein
SMU.72	-4.3	conserved hypothetical protein
SMU.1545c	-4.1	conserved hypothetical protein

SMU.502	-4.1	conserved hypothetical protein
SMU.36	-3.7	conserved hypothetical protein (eukaryotic-like)
SMU.1876	-2.9	conserved hypothetical protein
SMU.530c	-2.8	conserved hypothetical protein
SMU.721	-2.8	conserved hypothetical protein
SMU.1026	-2.8	conserved hypothetical protein
SMU.1701c	-2.7	conserved hypothetical protein
SMU.1377c	-2.6	conserved hypothetical protein
SMU.434	-2.6	conserved hypothetical protein
SMU.1436c	-2.6	conserved hypothetical protein
SMU.720	-2.5	conserved hypothetical protein
SMU.547	-2.5	conserved hypothetical protein
SMU.1582c	-2.5	conserved hypothetical protein
SMU.428	-2.5	conserved hypothetical protein, Cof family
SMU.1482c	-2.5	conserved hypothetical protein
SMU.1211	-2.4	conserved hypothetical protein
SMU.898	-2.4	conserved hypothetical protein
SMU.2059c	-2.4	conserved hypothetical protein; possible membrane protein
SMU.1414c	-2.4	conserved hypothetical protein
SMU.227c	-2.4	conserved hypothetical protein
SMU.2066c	-2.4	conserved hypothetical protein (possible integral membrane protein, ABC transporter)
SMU.1484c	-2.4	conserved hypothetical protein
SMU.1375c	-2.4	conserved hypothetical protein
SMU.1505c	-2.3	conserved hypothetical protein (phenylalanyl-tRNA synthetase fragment)
SMU.1853	-2.3	conserved hypothetical protein
SMU.1483c	-2.3	conserved hypothetical protein (possible acetyltransferase)
SMU.243	-2.3	conserved hypothetical protein
SMU.343	-2.3	conserved hypothetical protein
SMU.1314	-2.3	conserved hypothetical protein
SMU.1321c	-2.3	conserved hypothetical protein
SMU.543	-2.3	conserved hypothetical protein
SMU.1319c	-2.3	conserved hypothetical protein
SMU.471	-2.3	conserved hypothetical protein
SMU.1703c	-2.3	conserved hypothetical protein
SMU.1871c	-2.2	conserved hypothetical protein
SMU.2162c	-2.2	conserved hypothetical protein
SMU.1284c	-2.2	conserved hypothetical protein
SMU.2147c	-2.2	conserved hypothetical protein

SMU.1968c	-2.2	conserved hypothetical protein
SMU.239c	-2.2	conserved hypothetical protein
SMU.521	-2.1	conserved hypothetical protein
SMU.393	-2.1	conserved hypothetical protein
SMU.341	-2.1	conserved hypothetical protein (possible deoxyribonuclease)
SMU.523	-2.1	conserved hypothetical protein, VanZ-like family
SMU.737	-2.1	conserved hypothetical protein (possible alpha / beta superfamily hydrolase)
SMU.156	-2.1	conserved hypothetical protein
SMU.1773c	-2.1	conserved hypothetical protein
SMU.948	-2.1	conserved hypothetical protein
SMU.848	-2.1	conserved hypothetical protein
SMU.1406c	-2.0	conserved hypothetical protein (possible oxidoreductase)
SMU.1290c	-2.0	conserved hypothetical protein
SMU.1109c	-2.0	conserved hypothetical protein
SMU.159	-2.0	conserved hypothetical protein
SMU.1546	-2.0	conserved hypothetical protein
SMU.1140c	-2.0	conserved hypothetical protein (possible myo-inositol-1(or 4)-monophosphatase)
SMU.1704	-2.0	conserved hypothetical protein
SMU.392c	-2.0	conserved hypothetical protein, HI0933-like
SMU.1036	-2.0	conserved hypothetical protein
SMU.1171c	-2.0	conserved hypothetical protein
SMU.1392c	-2.0	conserved hypothetical protein; possible acetyltransferase
SMU.1347c	-2.0	Permease-ABC-type antimicrobial peptide transport system, Permease component
SMU.333	-2.0	conserved hypothetical protein
SMU.125	-2.0	conserved hypothetical protein
SMU.1153c	-2.0	conserved hypothetical protein
SMU.1784c	-2.0	conserved hypothetical protein, peptidase family M50
SMU.497c	-2.0	conserved hypothetical protein
SMU.475	-2.0	conserved hypothetical protein
SMU.751	-2.0	conserved hypothetical protein (possible transcriptional regulator)
SMU.407	-2.0	conserved hypothetical protein
SMU.1052	2.1	conserved hypothetical protein
SMU.1196c	2.8	conserved hypothetical protein
SMU.63c	3.3	conserved hypothetical protein
SMU.1072c	-7.6	acyltransferase
SMU.1450	-3.0	amino acid permease

SMU.1511c	-2.7	acetyltransferase; possible transcriptional repressor
SMU.546	-2.7	GTP-binding protein (tyrosine phosphorylated protein A); possible elongation factor
SMU.1434c	-2.6	glycosyl transferase
SMU.701c	-2.5	integral membrane protein
SMU.623c	-2.5	polysaccharide deacetylase
SMU.1061	-2.4	DNA-binding protein
SMU.1700c	-2.3	LrgB-like protein; possible murein hydrolase regulator
SMU.1100c	-2.3	permease
SMU.2043c	-2.2	D-tyrosyl-tRNA deacylase
SMU.1444c	-2.2	metallo-beta-lactamase superfamily proteinhetical protein
SMU.368c	-2.2	metallo-beta-lactamase superfamily protein
SMU.348	-2.1	histidine triad (HIT) hydrolase
SMU.1558c	-2.1	acetyltransferase, GNAT family
SMU.1969c	-2.1	probable transcriptional regulator
SMU.1555c	-2.0	ribonuclease BN-like family protein
SMU.337	-2.0	inner membrane protein
SMU.1702c	-2.0	uncharacterized phosphatase; possible PAP2 family protein
SMU.1029	-9.2	hypothetical - transposon
SMU.189	-2.6	hypothetical protein
