# Supplementary material for: Transcriptional Profiling of the Oral Pathogen Streptococcus mutans in Response to Competence Signaling Peptide XIP
Source: mSystems. 2017 Jan 3;2(1):e00102-16. doi: 10.1128/mSystems.00102-16 (PMC5209530; doi:10.1128/mSystems.00102-16)
Supplement: TABLE S3 [file sys001172075st3.pdf]

Table S3. Genes up- or down-regulated in ΔSMcomS in response to 1 µM XIP.

Gene ID	Fold Change	Annotation
**Cell killing and bacteriocin production:**		
SMU.1910c	4.6	hypothetical protein
SMU.1912c	4.0	hypothetical protein
SMU.1913c	3.0	putative immunity protein; BlpL-like; immA
SMU.1914c	3.1	hypothetical protein; nlmC; mutacin V
SMU.1908c	4.2	hypothetical protein; immunity protein
SMU.1909c	5.1	hypothetical protein
SMU.1902c	6.5	hypothetical protein
SMU.1903c	6.0	putative bacteriocin secretion protein; bsmL
SMU.1904c	5.5	hypothetical protein
SMU.1905c	5.0	hypothetical protein
SMU.1906c	6.5	bacteriocin-related protein
SMU.150	2.0	nlmA; non-lantibiotic mutacin IV A
SMU.151	2.1	nlmB; non-lantibiotic mutacin IV B
SMU.152	2.3	hypothetical protein
SMU.423	4.0	nlmD; possible bacteriocin
SMU.836	71.0	hypothetical protein
SMU.299c	3.4	ip; bacteriocin peptide precursor
SMU.1917	7.8	comE; response regulator
SMU.1916	8.9	comD; histidine kinase
SMU.1915	2.2	comC, competence stimulating peptide
SMU.168	5.9	transcriptional regulator toxin-antitoxin
SMU.166	5.4	putative toxin-antitoxin system
SMU.167	6.4	putative toxin-antitoxin system
**Competence and DNA transformation:**		
SMU.1001	525.1	dprA; DNA processing protein; Smf family
SMU.1981c	936.7	competence protein comG
SMU.1983	408.9	competence protein comYD
SMU.1984	1185.2	competence protein comYC
SMU.1997	61.5	comX; competence-specific sigma factor
SMU.498	351.0	comFA; late competence protein F
SMU.625	183.8	comEA; competence protein
SMU.626	394.6	comEC; competence protein
SMU.499	277.3	comFC; late competence protein
SMU.644	216.0	coiA; competence protein
SMU.1987	625.8	comGA; late competence protein

SMU.2086	10.0	cinA; competence damage-inducible protein A
SMU.1985	783.0	comGB; competence protein
**DNA metabolism, replication, recombination and repair:**		
SMU.02	2.4	dnaN; DNA polymerase III, beta subunit
SMU.1002	8.9	topA; DNA topoisomerase I
SMU.1055	27.5	radC; DNA repair protein
SMU.1056	3.4	DNA repair protein RadC
SMU.1174	2.8	pcrA; ATP-dependent DNA helicase
SMU.1192	2.4	dnaE; DNA-polymerase III subunit alpha
SMU.123	2.2	polC; DNA-polymerase III, alpha subunit
SMU.1472	3.0	recJ; single-strand DNA-specific exonuclease
SMU.1500	2.0	rexB; ATP-dependent exonuclease subunit B
SMU.1714c	2.0	xerD; tyrosine recombinase
SMU.1967	215.1	ssbA; single-stranded DNA-binding protein
SMU.2085	6.5	recA; recombinase A
SMU.505	9.4	dpn; adenine-specific DNA methylase (DpnIIB)
SMU.506	7.4	ssuRB; type II restriction endonuclease
SMU.64	12.0	ruvB; holiday junction DNA helicase
SMU.821	2.1	dnaG; DNA primase
SMU.510c	-2.5	deoxyribonuclease
**Transport and binding:**		
SMU.109	2.2	lantibiotic related antibiotic efflux protein/macrolide permease
SMU.1658	-3.5	ammonium transporter, NrgA protein
SMU.770c	8.0	manganese transporter/possible HitA ferric iron-binding periplasmic protein
SMU.1013c	2.3	Mg2+/citrate complex transporter
SMU.980	3.2	beta-glucoside-specific EII permease
SMU.772	8.9	bifunctional glucan-binding protein D and lipase
SMU.263	2.8	amino acid permease /putrescine antiporter
SMU.1568	-3.8	maltose / maltodextrin-binding protein
SMU.976	1.9	spermidine/putrescine ABC transporter, spermidine/putrescine-binding protein
SMU.1900	2.1	ABC transporter
SMU.998	2.4	ABC transporter, ferrichrome-binding protein
SMU.933	2.8	amino acid ABC transporter, amino acid substrate-binding protein
SMU.1963c	3.5	sugar-binding periplasmic protein
SMU.1966c	4.3	levT, ABC transport ribose-binding protein, periplasmic
SMU.1570	-3.1	maltose / maltodextrin ABC transport system

		(permease)
SMU.1569	-2.7	maltodextrin ABC transport system permease
SMU.2160	2.0	possible permease, ABC transporter protein
SMU.909	2.0	malate permease
SMU.1093	2.1	ABC transporter permease protein
SMU.934	2.1	amino acid ABC transporter, permease protein
SMU.183	2.2	manganese ABC transporter permease element
SMU.1149	2.2	ABC transporter, membrane spanning
SMU.657	2.3	ABC transporter, permease, possibly bacteriocin associated
SMU.864	2.4	ABC transporter, permease component
SMU.996	2.4	ABC transporter, permease protein;possible ferrichrome transport system
SMU.1928	2.4	protein secretion ABC transport permease
SMU.975	2.4	spermidine/putrescine ABC transporter, permease
SMU.653c	2.5	ABC transporter, permease protein (possible taurine transport system permease)
SMU.1067c	2.6	ABC transporter, permease protein
SMU.906	2.9	ABC transporter, ATP-binding / permease protein
SMU.656	3.3	ABC transporter, permease, possibly bacteriocin associated
SMU.1166c	3.4	ABC transporter permease
SMU.905	2.8	ABC-type multidrug/protein/lipid transport system, ATPase component
SMU.2116	-2.1	glycine betaine / carnitine / choline ABC transporter, ATP-binding protein, opuCA
SMU.654	2.0	ABC transporter, ATP-binding protein
SMU.1551c	2.0	ABC transporter, ATP-binding protein
SMU.997	2.2	inorganic ion ABC transporter,ATP-binding protein; possible ferrichrome transport system
SMU.1068c	2.2	ABC transporter, ATP-binding protein
SMU.1041	2.4	ABC transporter, ATP-binding protein
SMU.863	2.4	ABC transporter, ATP-binding protein
SMU.1927	2.8	PsaA protein/ ABC transporter, ATP-binding protein
SMU.1148	2.8	ABC transporter, ATPase component
SMU.1167c	3.1	ABC transporter ATP-binding protein
SMU.1899	4.4	ABC transport fragment
**Signal transduction and transcriptional regulation:**		
SMU.1926	2.0	transcriptional regulator PsaR
SMU.1168	2.0	transcriptional regulator (TetR/AcrR family)
SMU.977	2.6	transcription antiterminator LicT (fragment)
SMU.1977c	2.8	transcriptional regulator

SMU.1995c	3.1	zinc transport transcriptional repressor
SMU.261c	3.4	transcriptional regulator
SMU.592c	3.9	transcriptional regulator
SMU.507	4.9	transcriptional regulator, DeoR family
SMU.514	6.6	transcriptional regulator, AcrR family
SMU.1733c	2.1	SNF helicase
SMU.1474c	2.8	ribonuclease Z
SMU.994	2.2	ribonuclease HII
SMU.1517	2.0	vicR, two-component response regulator
SMU.1516	2.0	vicK, two-component sensor histidine kinase
SMU.1964c	3.9	levR, two-component response regulator
SMU.1965c	4.1	levS, histidine kinase
SMU.1877	-2.0	manL, mannose PTS system component IIAB
SMU.674	-2.8	phosphotransferase system phosphohistidine-containing protein
SMU.1600	-2.2	PTS system IIB component, required for cellobiose uptake and metabolism
SMU.1958c	2.8	levF, fructose-specific Enzyme IIC component
SMU.1960c	3.0	levE, fructose-specific Enzyme IIB component
SMU.1957	3.3	levG, fructose-specific Enzyme IID component
SMU.1961c	3.4	levD, fructose-specific Enzyme IIA component
SMU.65	11.7	protein tyrosine-phosphatase
SMU.1515	2.4	vicX, gtfB/C regulator, metallo-beta-lactamase superfamily
SMU.1053	10.5	possible redox-sensing transcriptional repressor Rex
SMU.1919	2.3	sakacin A production response regulator
SMU.363	-2.8	glnR, transcriptional regulator; glutamine synthetase repressor
**Energy metabolism:**		
SMU.671	-4.2	citrate synthase
SMU.670	-4.0	aconitate hydratase; aconitase A
SMU.672	-3.9	isocitrate dehydrogenase
SMU.1495	2.0	galactose-6-phosphate isomerase
SMU.981	3.0	beta-glucosidase, BglB protein
SMU.2028	4.2	fructosyltransferase
SMU.982	4.2	beta-glucosidase, BglB protein
SMU.646	13.6	hydrolase (possible phosphoglycolate phosphatase)
SMU.2142	3.3	sugar-phosphate isomerase (ribose 5-phosphate isomerase)
SMU.352	4.0	ribulose-phosphate-3-epimerase
SMU.1170	2.3	cytochrome c-type biogenesis protein

SMU.1043c	2.0	phosphate acetyltransferase
SMU.1422	2.1	acetoin dehydrogenase E1 component
SMU.1423	2.8	acetoin dehydrogenase E1 component
SMU.1421	2.9	dihydrolipoamide acetyltransferase (acetoin dehydrogenase E2 component)
SMU.1424	3.7	dihydrolipoamide dehydrogenase
SMU.1978	17.9	acetate kinase
SMU.665	2.5	acetylglutamate kinase
**Central intermediary metabolism:**		
SMU.132	2.2	amino acid amidohydrolase (hippurate amidohydrolase)
SMU.1322	4.2	acetoin reductase
SMU.664	3.0	ornithine acetyltransferase / N-acetylglutamate synthase
SMU.663	3.2	N-acetyl-gamma-glutamyl-phosphate reductase (N-acetyl-glutamate-gamma-semialdehyde dehydrogenase)
SMU.264	2.0	agmatine deiminase
**Fatty acid and phospholipid metabolism:**		
SMU.1335c	2.1	enoyl-acyl carrier protein(ACP) reductase; dioxygenase related to 2-nitropropane dioxygenase
**Cell division:**		
SMU.1713c	2.0	segregation and condensation protein A
SMU.1003	10.2	glucose-inhibited division protein
**Pathogenesis, toxin production and resistance:**		
SMU.515	7.3	mycA; 67 kDa myosin-crossreactive antigen
SMU.610	-6.0	spaP; cell surface antigen
SMU.1340	2.0	bacA; bacitracin synthetase 1/ tyrocidin synthetase III
**Adaptation to atypical conditions and detoxification:**		
SMU.1046c	2.8	relQ; relA yjbM; GTP pyrophosphokinase
SMU.1286c	2.9	multidrug resistance permease
SMU.1338c	2.2	mefE; ABC transport macrolide permease
**Mobile and extrachromosomal element functions:**		
SMU.1398	2.4	irvR; repressor protein - phage associated
SMU.1329c	2.4	paaB; transposase fragment
SMU.1351	2.0	transposase fragment
SMU.1372c	2.0	transposase, IS861, IS3 family
SMU.149	2.0	transposase fragment (IS605/IS200-like)

SMU.590c	4.0	transposase fragment
SMU.1024c	2.0	transposase fragment
SMU.767	2.6	transposase
SMU.1380	3.1	Pseudo-Spn1 transposase
SMU.766	4.1	transposase
**Purines, pyrimidines, nucleosides, and nucleotides:**		
SMU.1054	20.3	guaA; glutamine amidotransferase
SMU.29	-4.2	purC; phosphoribosylaminoimidazole-succinocarboxamide synthase
SMU.30	-4.6	purL; phosphoribosylformylglycinamidine synthase
SMU.32	-3.7	purB; amidophosphoribosyltransferase
SMU.34	-3.3	purM; phosphoribosylformylglycinamide cyclo-ligase (AIRS)
SMU.35	-2.4	purN; phosphoribosylglycinamide formyltransferase
SMU.37	-2.5	purH; phosphoribosylaminoimidazolecarboxamide formyltransferase / IMP cyclohydrolase
SMU.48	-2.6	purD; phosphoribosylamine-glycine ligase
SMU.50	-2.7	purE; phosphoribosylaminoimidazole carboxylase catalytic subunit
SMU.51	-3.0	purK; phosphoribosylaminoimidazole carboxylase, ATPase subunit
SMU.356	4.0	purR; purine operon repressor/xanthine phosphoribosyltransferase
**Amino acid biosynthesis:**		
SMU.532	2.6	trpE; anthranilate synthase, component I
SMU.533	4.4	trpG; anthranilate synthase, component II
SMU.534	2.7	trpD; phosphoribosyl anthranilate transferase
SMU.535	4.1	trpC; indole-3-glycerol phosphate synthase
SMU.536	6.1	trpF; phosphoribosylanthranilate isomerase
SMU.537	6.2	trpB; tryptophan synthase, beta subunit
SMU.538	15.8	trpA; tryptophan synthase, alpha subunit
SMU.1657c	-2.5	glnB; nitrogen regulatory protein PII
SMU.364	-2.7	glnA; glutamine synthetase type 1
SMU.1381	2.9	leuD; 3-isopropylmalate dehydratase, small subunit
SMU.1382	3.0	leuC; alpha-isopropylmalate isomerase large subunit
SMU.1383	2.6	leuB; 3-isopropylmalate dehydrogenase
SMU.1384	2.6	leuA; 2-isopropylmalate synthase
SMU.1173	2.6	cysD; O-acetylhomoserine sulfhydrylase
SMU.1920	2.2	pdgA; phosphoglycerate dehydrogenase
SMU.666	2.4	N-acetylornithine aminotransferase
SMU.262	4.9	ornithine carbamoyltransferase

SMU.54	-2.4	amino acid racemase
**Biosynthesis of cofactors, prosthetic groups, and carriers:**		
SMU.838	5.8	gor; glutathione reductase
SMU.1045c	2.6	ppnK; NAD(+) kinase (ATP-NAD kinase)
SMU.353	4.2	thiamine pyrophosphokinase
**Cell envelope, biosynthesis and degradation of murein sacculus and peptidoglycan:**		
SMU.21	2.0	cell shape-determining protein MreD
**Protein synthesis and protein fate:**		
SMU.1132	2.0	pepN; lysyl-aminopeptidase
SMU.539c	45.1	prepilin peptidase type IV
SMU.645	15.6	PepB oligopeptidase
SMU.1610	3.6	rpmG; 50S ribosomal protein L33
SMU.340	2.6	rpmH; 50S ribosomal protein L34
SMU.500	2.1	yfiA; ribosome-associated protein
SMU.1044c	2.6	rluE; ribosomal large subunit pseudouridine synthase
SMU.2143c	2.6	trmU; tRNA (5-methylaminomethyl-2-thiouridylate)-methyltransferase
SMU.r06	-3.0	
SMU.r03	-3.0	
SMU.r05	-2.4	
SMU.r02	-2.3	
**tRNA:**		
SMU.t22	-4.6	
SMU.t03	-3.6	
SMU.t02	-3.4	
SMU.t07	-3.0	
SMU.t21	-2.9	
SMU.t01	-2.9	
SMU.t61	-2.8	
SMU.t05	-2.3	
SMU.t42	2.2	
SMU.t30	2.3	
SMU.t09	2.4	
SMU.t37	2.4	
SMU.t59	2.5	
SMU.t08	2.6	
SMU.t63	3.6	

**Other:**		
SMU.1073	-2.5	formate--tetrahydrofolate ligase
SMU.31	-4.3	conserved hypothetical protein
SMU.1403c	-2.7	conserved hypothetical protein
SMU.1405c	-2.6	conserved hypothetical protein
SMU.53	-2.5	conserved hypothetical protein
SMU.1968c	-2.5	conserved hypothetical protein
SMU.36	-2.4	conserved hypothetical protein (eukaryotic-like)
SMU.673	-2.4	conserved hypothetical protein
SMU.502	-2.3	conserved hypothetical protein
SMU.1764c	-2.3	conserved hypothetical protein
SMU.1402c	-2.3	conserved hypothetical protein
SMU.1803c	-2.3	conserved hypothetical protein
SMU.1404c	-2.1	conserved hypothetical protein
SMU.52	-2.1	conserved hypothetical protein
SMU.1763c	-2.0	conserved hypothetical protein
SMU.560c	-2.0	conserved hypothetical protein
SMU.845	2.0	conserved hypothetical protein
SMU.345c	2.0	conserved hypothetical protein (possible transcription regulator)
SMU.844	2.0	conserved hypothetical protein
SMU.1923c	2.0	conserved hypothetical protein
SMU.1956c	2.0	conserved hypothetical protein
SMU.1701c	2.0	conserved hypothetical protein
SMU.807	2.1	conserved hypothetical protein (possible membrane protein)
SMU.746c	2.1	conserved hypothetical protein
SMU.1048	2.1	conserved hypothetical protein
SMU.523	2.1	conserved hypothetical protein, VanZ-like family
SMU.173	2.1	conserved hypothetical protein (possible ppGpp-regulated growth inhibitor)
SMU.1080c	2.1	conserved hypothetical protein
SMU.1642c	2.1	conserved hypothetical protein
SMU.929c	2.2	conserved hypothetical protein
SMU.217c	2.2	conserved hypothetical protein; Streptococcus-specific protein
SMU.1336	2.2	conserved hypothetical protein
SMU.290	2.2	conserved hypothetical protein
SMU.627	2.2	conserved hypothetical protein
SMU.1442c	2.2	conserved hypothetical protein
SMU.473	2.3	conserved hypothetical protein

SMU.1678	2.4	conserved hypothetical protein
SMU.835	2.4	conserved hypothetical protein (possible membrane protein)
SMU.820	2.4	conserved hypothetical protein
SMU.1070c	2.4	conserved hypothetical protein
SMU.1975c	2.4	conserved hypothetical protein
SMU.1172c	2.5	conserved hypothetical protein
SMU.757	2.5	conserved hypothetical protein
SMU.1856c	2.5	conserved hypothetical protein
SMU.1976c	2.6	conserved hypothetical protein
SMU.1042	2.6	conserved hypothetical protein; possible ABC transporter, permease
SMU.1884c	2.8	conserved hypothetical protein
SMU.1280c	2.9	conserved hypothetical protein (possible alpha/beta superfamily hydrolase)
SMU.2084c	2.9	conserved hypothetical protein (possible arsenate reductase)
SMU.630	3.1	conserved hypothetical protein
SMU.1400c	3.1	conserved hypothetical protein
SMU.1475c	3.1	conserved hypothetical protein
SMU.631	3.2	conserved hypothetical protein
SMU.932	3.2	conserved hypothetical protein
SMU.1621c	3.2	conserved hypothetical protein
SMU.591c	3.2	conserved hypothetical protein
SMU.1284c	3.6	conserved hypothetical protein
SMU.790	3.7	conserved hypothetical protein
SMU.354	4.5	conserved hypothetical protein
SMU.508	4.5	conserved hypothetical protein (possible hydrolase)
SMU.2080	5.0	brsR, conserved hypothetical protein
SMU.66	6.9	conserved hypothetical protein; possible phosphatidylinositol-4-phosphate 5-kinase
SMU.63c	8.5	conserved hypothetical protein
SMU.769	19.2	conserved hypothetical protein
SMU.1979c	92.5	conserved hypothetical protein, methyltransferase domain
SMU.1982c	715.7	conserved hypothetical protein
SMU.1980c	1618.5	conserved hypothetical protein
SMU.1969c	-2.6	probable transcriptional regulator
SMU.987	-2.0	cell wall surface anchor family protein
SMU.679	2.0	oxidoreductase, aldo/keto reductase family
SMU.834	2.1	glycosyltransferase
SMU.1476c	2.1	GTP-binding protein
SMU.1337c	2.1	alpha/beta superfamily hydrolases

SMU.641	2.3	oxidoreductase
SMU.574c	2.3	effector of murein hydrolase
SMU.346	2.4	NADH dehydrogenase; NAD(P)H nitroreductase
SMU.1918	2.4	membrane-associated protein DedA
SMU.1169c	2.5	thioredoxin family protein
SMU.1996	2.5	isopentenyl monophosphate kinase (4-diphosphocytidyl-2-C-methyl-D-erythritol kinase)
SMU.993	2.6	GTP-binding protein
SMU.1473c	2.8	oxidoreductase, short chain dehydrogenase/reductase family
SMU.643	3.3	acetyl esterase/ sugar hydrolase
SMU.647	3.4	O-methyltransferase
SMU.355	4.9	CMP-binding factor 1
SMU.67	5.8	acyltransferase
SMU.837	29.7	oxidoreductase, aldo/keto reductase family
SMU.1895c	-5.2	hypothetical protein
SMU.1896c	-4.3	hypothetical protein
SMU.18	-4.1	hypothetical protein
SMU.1804c	-3.8	hypothetical protein
SMU.33	-3.6	hypothetical protein
SMU.1000	-3.0	hypothetical protein
SMU.278	-2.7	hypothetical protein
SMU.281	-2.6	hypothetical protein
SMU.279	-2.5	hypothetical protein
SMU.1752c	-2.5	hypothetical protein
SMU.503c	-2.4	hypothetical protein
SMU.501	-2.3	hypothetical protein
SMU.49	-2.3	hypothetical protein
SMU.284	-2.3	hypothetical protein
SMU.958	-2.2	hypothetical protein
SMU.55	-2.1	hypothetical protein
SMU.277	-2.0	hypothetical protein
SMU.1399	-2.0	hypothetical protein
SMU.594	-2.0	hypothetical protein
SMU.214c	2.0	hypothetical protein
SMU.739c	2.0	hypothetical protein
SMU.748	2.1	hypothetical protein
SMU.1855	2.1	high density responsive membrane protein, HdrM
SMU.1369	2.1	hypothetical protein
SMU.1256c	2.1	hypothetical protein
SMU.813	2.1	hypothetical protein (possible transcriptional regulator)
SMU.185	2.1	hypothetical protein

SMU.1047c	2.1	hypothetical protein
SMU.1504c	2.1	hypothetical protein
SMU.216c	2.1	hypothetical protein
SMU.811	2.2	hypothetical protein
SMU.1310	2.2	hypothetical protein
SMU.219	2.2	hypothetical protein
SMU.1069c	2.2	hypothetical protein
SMU.215c	2.2	hypothetical protein
SMU.642	2.2	hypothetical protein
SMU.1373c	2.3	hypothetical protein
SMU.1891c	2.3	hypothetical protein
SMU.176	2.3	hypothetical protein
SMU.1862	2.4	hypothetical protein
SMU.1091	2.6	hypothetical protein (possible cell wall protein)
SMU.220c	2.6	hypothetical protein
SMU.379	2.7	hypothetical protein
SMU.1553c	2.8	hypothetical protein
SMU.992	2.8	hypothetical protein
SMU.2083c	3.1	hypothetical protein
SMU.791c	3.1	hypothetical protein
SMU.812	3.4	hypothetical protein
SMU.1368	3.9	hypothetical protein
SMU.2081	4.2	brsM, hypothetical protein
SMU.513	5.1	hypothetical protein
SMU.68	5.5	hypothetical protein
SMU.1907	5.6	hypothetical protein
SMU.2076c	6.5	hypothetical protein
SMU.771c	7.1	hypothetical protein
SMU.1378	10.3	hypothetical protein
SMU.189	-5.6	hypothetical protein
SMU.93c	-2.2	hypothetical protein
SMU.2053c	2.4	hypothetical protein (peptide)
SMU.437c	2.7	Pseudo gene
SMU.1379	3.7	hypothetical protein-HTH XRE domain
SMU.41	6.0	hypothetical protein
